# Neurological sequel of chronic kidney disease: From diminished Acetylcholinesterase activity to mitochondrial dysfunctions, oxidative stress and inflammation in mice brain

**DOI:** 10.1038/s41598-018-37935-3

**Published:** 2019-02-28

**Authors:** Muhammed Khairujjaman Mazumder, Rajib Paul, Pallab Bhattacharya, Anupom Borah

**Affiliations:** 1 Cellular and Molecular Neurobiology Laboratory, Department of Life Science and Bioinformatics, Assam University, Silchar - 788011, Assam India; 2 Department of Zoology, Pandit Deendayal Upadhyaya Adarsha Mahavidyalaya (PDUAM), Eraligool-788723, Karimganj, Assam India; 3 Department of Pharmacology and Toxicology, National Institute of Pharmaceutical Education and Research (NIPER)-Ahmedabad, Gandhinagar - 382355, Gujarat India

## Abstract

With increasing prevalence, chronic kidney disease (CKD) has become a global health problem. Due to the retention of uremic toxins, electrolytes and water, and the resultant metabolic disturbances, CKD affects several organs, including the nervous system. Thus, CKD patients suffer from several neurological complications, including dementia, cognitive impairment, motor abnormalities, depression, and mood and sleep disturbances. However, the mechanisms underlying the neurological complications are least elucidated. We have recently reported a highly reproducible mice model of CKD induced by high adenine diet, which exhibited psychomotor behavioral abnormalities and blood-brain barrier disruption. In the present study, using the mice model, we have investigated psycho-motor and cognitive behaviour, and the neurochemical and histopathological alterations in brain relevant to the observed behavioural abnormalities. The results demonstrate global loss of Acetylcholinesterase activity, and decrease in neuronal arborisation and dendritic spine density in discrete brain regions, of the CKD mice. Oxidative stress, inflammation, and mitochondrial dysfunctions were found in specific brain regions of the mice, which have been regarded as the underlying causes of the observed neurochemical and histopathological alterations. Thus, the present study is of immense importance, and has therapeutic implications in the management of CKD-associated neurological complications.

## Introduction

Chronic kidney disease (CKD) refers to a wide spectrum of disease conditions whereby the renal structure and functions are impaired. This leads to decrease in the glomerular filtration rate below 60 mL/min/1.73 m^2^, and the resultant retention of uremic toxins in the body^1,2^. CKD is a global health issue, affecting more than 15% of the adult population in developed nations^3,4^. In India, the overall prevalence is 17.2%^5,6^, while in some coastal districts of Andhra Pradesh (India), it has been reported to be more than 60%^7^. Thus, CKD has become a major health burden, especially for the developing countries lacking sufficient professionals and infrastructure.

Once renal damage is initiated, factors including proteinuria, hyperglycemia, hypertension, metabolic disturbances, and lifestyle factors like smoking, dehydration and low fiber intake contribute to the progression of the disease to end-stage renal disease^8,9^. In the end stages of the disease, anaemia, low levels of serum albumin and high phosphate increase morbidity and mortality^1,9^. Moreover, with progression of the disease, and retention of metabolic wastes, electrolytes and water in the body, CKD leads to edema, cardiac failure, arrhythmia, bone disease, changes in pigmentation, insulin resistance, thiamine and calciferol deficiency, liver infection, dyslipidemia and hyperhomocysteinemia^10–13^. With reduction in glomerular filtration rate, and consequent retention of uremic toxins, and the associated disturbances^10–13^, CKD affects other organs^14,15^, including the nervous system, which results in neurological complications^16–18^.

The CKD patients suffer from several neurological complications, including anxiety, depression, motor abnormalities (restless-leg syndrome; RLS), sleep disturbances and cognitive dysfunctions^17–21^. In children with CKD, reduced intelligence quotient, memory, and language and academic achievements have been reported^22^. We have recently reported psychomotor behavioral abnormalities and blood-brain barrier disruption in mice model of the disease^23^. Cognitive decline, both acute and chronic with dementia, increases with advancements in the severity of the disease, and may affect 80% of the subjects^17,24,25^. Cognitive decline is known to be caused due to cholinergic deficiency, including decrease in the activity of Acetylcholinesterase (AChE) in brain^26,27^. However, the mechanism underlying cognitive decline in CKD, and the role of AChE thereto, has not been investigated so far. RLS is a motor behavioral abnormality with a prevalence of 15–20% among CKD patients^20^, and is also associated with sleep disturbances^28^. Moreover, motor behavioral abnormalities similar to parkinsonism, including resting tremor, rigidity, bradykinesia and postural instability, have been reported in CKD patients^29^. Dopamine agonist and levodopa therapy are practised for the amelioration of RLS^30^. However, the effect of CKD on the dopaminergic neurons of the nigrostriatum has not been investigated yet.

In mice model of acute renal injury, hyperactivation of glia (astroglia) has been reported^31^. However, similar study in CKD model has not been undertaken. Moreover, CKD-induced possible oxidative stress, inflammation, mitochondrial dysfunctions, and dendritic atrophy and loss of dendritic spine density in the brain, which may be the pathophysiological basis of the neurological complications, remain largely unknown. Thus, the present study was undertaken to elucidate the biochemical and histopathological changes relevant to the neurological complications in CKD.

## Materials and Methods

### Animals

Swiss Albino male mice of weight between 25–27 g (aged 6–7 weeks) were purchased from the animal house of the Pasteur Institute, Shillong, Meghalaya (India). They were maintained under standard laboratory conditions, and were given standard feed and water *ad libitum*. The experimental protocols used in the present study have been approved by the Animal Ethics Committee, Assam University, Silchar, India (IEC/AUS/2013-055, dated 20/03/2013). All the protocols used in the present study were performed in accordance with the relevant guidelines and regulations.

### Chemicals and others

Acetylthiocholine iodide, adenine, ketamine, xylazine, thiobarbituric acid, 1,1,3,3-tetramethoxypropane, 3,3-diaminobenzidine (DAB) liquid substrate system, hydrogen peroxide (H_2_O_2_), Sirius red and poly-L-lysine were obtained from Sigma-Aldrich Co., USA. Potassium dichromate, silver nitrate and formaldehyde were obtained from HiMedia laboratories Pvt. Ltd., India. Acetic acid, cresyl violet, Nitro blue tetrazolium (NBT), 5,5′-dithiobis-(2-nitrobenzoic acid) (DTNB), cytochrome c, pyrogallol, salicylic acid, tri-sodium citrate, copper sulphate, potassium ferricyanide, paraformaldehyde, picric acid, Triton X-100, sodium dodecyl sulphate and other chemicals were of analytical reagent grade, procured from SISCO Research Laboratories Pvt. Ltd., India. Rabbit anti-Glial fibrillary acidic protein (GFAP) antibody (ab7260) and rabbit anti-tyrosine hydroxylase (TH) antibody (ab112) were procured from Abcam (Cambridge, UK). Anti-rabbit goat secondary antibody conjugated with horseradish peroxidase (HRP; ap307p) was procured from Millipore Co. (USA). Urea, creatinine and uric acid assay kits were obtained from Coral Clinical Systems, Goa, India (Reference no. 1102240075, 1101070275, 1102260275).

### Experimental design

Following acclimatization of 10 days, the animals were randomly divided into 2 groups (n = 42 each): the first group served as control and was given standard powdered diet, and the second group (CKD group) received adenine-rich diet, at 0.3% w/w mixed with the feed, for 4 weeks. Body weights of the mice were taken on every alternative day. At the end of the treatment period, six mice from each group were subjected to motor behavioural test (Swim test), following which they were sacrificed. The mice were anaesthetised with ketamine (75 mg/kg b.w.) and xylazine (5 mg/kg b.w.), blood was collected by cardiac puncture (for estimation of urea, creatinine and uric acid), and then they were perfused transcardially. Kidneys and brains were harvested and processed for renal histology using Hematoxylin-Eosin staining, and brain histology and immunohistochemistry. Another set of mice (n = 6 each) were used for psychometric behavioural test (Forced Swim test), after which they were perfused with glycerol, brains were harvested, and were used for histoenzymological studies of AChE, mitochondrial complexes (II and III) and neuronal nitric oxide synthase (nNOS). Another group of mice (n = 24) were sacrificed by decapitation, brains were harvested, different brain regions were isolated, homogenised and were used for estimation of the activities of Acetylcholinesterase, mitochondrial complex-I, superoxide dismutase and catalase, and lipid peroxidation. Rest of the mice (n = 6 each) were used for Cognitive behavioural test, and then sacrificed for Rapid Golgi staining. All the behavioural tests and histological studies, including scoring, were performed by assessors blind to the experimental groups.

### CKD model generation

The mice model of CKD was developed by providing adenine-rich diet (0.3% w/w mixed with feed) for 4 weeks, as per the protocol standardised in our laboratory^23^. Serum urea, creatinine and uric acid levels, and renal histology were assessed for ascertaining establishment of CKD in the model. Body weights of the mice were taken every alternative day.

### Behavioural tests

#### Motor behavioural test

Swimming ability test was performed to determine motor performance, following Mazumder *et al*.^23^. The test was performed in plastic tubs of dimensions 40 cm × 25 cm × 16 cm, containing 12 cm water level, maintained at 27 ± 2 °C. The swim score of each minute, for a total of 10 min, was calculated on a scale of 0–3: ‘3’ – continuous swimming, ‘2’ – continuous swimming with occasional floating, ‘1’ –more floating and occasional swimming with hind limbs, and ‘0’ – hind parts sinking with only head floating. Water was changed after each test for each mice.

#### Psychometric behavioural test

Forced swim test was performed to determine depression-like behaviour in the mice, following Mazumder *et al*.^23^. The test was performed in glass cylinders of 25 cm height and 12 cm diameter, with 12 cm water level, maintained at 27 ± 2 °C. Mice were released in water, and following 2 min acclimatization, the total immobility time was recorded for each mice for a period of 4 min. Water was changed after each test for every mice.

#### Cognitive function test

Object location memory (OLM) and object recognition memory (ORM) tests of the experimental animals were performed to assess their cognitive functions. The tests were performed following Paul and Borah^32^. For performing OLM test, two identical objects were placed 7 cm apart from each other in a chamber of 30 cm × 23 cm dimensions. This rectangular chamber had 2 longer sides (30 cm each) and 2 shorter sides (23 cm each) opposite to each other respectively. The objects were placed 1 cm away from shorter sides and 6.5 cm away from longer sides of the chamber. Animals were placed in the opposite side of the object and 2.5 cm away from shorter wall of the chamber. Animals were trained in this setup for 10 min. After an interval of 90 min post training, test was done in which one of the objects was shifted to a new location. The exploration time for both the objects was recorded for 5 min to calculate the discrimination index (DI). As there was 90 min interval between training and test, OLM gives score for short-term memory.

ORM test was performed using a cylindrical chamber of 28 cm diameter. Objects were placed diagonally 8 cm apart from each other and 3 cm away from the circumference. On 4^th^ day following OLM test, ORM training of the animals was performed for 10 min each with similar objects. After an interval of 24 h following the training session, ORM test was performed for 5 min with a novel object and the exploration time in both the objects was recorded to calculate the DI. As there was 24 h interval between training and test, ORM gives score for short-term memory. The Discrimination Index (DI) is calculated as follows: DI = [(time exploring the novel object − time exploring the familiar)/(time exploring novel + familiar) × 100].

## Validation of CKD Model

The induction of CKD in mice was validated using haematological and renal histopathological changes.

### Hematological parameters

After the treatment period, the mice were anaesthetised by ketamine (75 mg/kg; i.p.) and xylazine (5 mg/kg; i.p.), and blood was collected in vacutainer vials containing clot activators. Serum was obtained following centrifugation at 2000 × g for 5 min using Refrigerated Centrifuge (Eppendorf, Germany), and was used for estimation of urea, creatinine and uric acid. Urea was assayed following modified Berthelot Method^33^, using commercially available enzymatic urea assay kit supplied in ready to use form. Briefly, urea in serum is hydrolysed by Urease enzyme to produce ammonia, which reacts with the phenolic chromogen and hypochlorite to form a green coloured complex, the intensity of which is directly proportional to the amount of urea present. Absorbance was measured at 570 nm using spectrophotometer (Thermo Fisher Scientific, Finland). The method is linear upto 250 mg/dL of urea in serum.

Serum creatinine level was estimated using alkaline picrate Jaffe’s reaction method^34^, using commercially available assay kit. Briefly, creatinine reacts with picric acid in alkaline medium to produce an orange coloured complex with alkaline picrate, the intensity of which is directly proportional to the amount of creatinine present. The coloured complex has peak absorbance at 520 nm, which was measured using spectrophotometer (Thermo Fisher Scientific, Finland). To minimize interference, deproteinization of serum was done by mixing 0.1 mL of serum with 1 mL of picric acid reagent, followed by centrifugation at 3000 rpm for 10 min to obtain a clear supernatant, which was used for the estimation of creatinine. The method is linear upto 20 mg/dL of creatinine in serum.

Estimation of uric acid was done following Uricase/PAP method^35^, using commercially available enzymatic assay kit. Briefly, uric acid is degraded by Uricase enzyme to allantoin and H_2_O_2_. H_2_O_2_ reacts with a phenolic compound and 4-aminoantipyrine by peroxidase to produce a red coloured quinoneimine dye complex, the intensity of which is directly proportional to the amount of uric acid present in the serum. Absorbance was recorded at 520 nm using spectrophotometer (Thermo Fisher Scientific, Finland). The method is linear upto 20 mg/dL of uric acid.

All reactions were carried out at 37 °C, and reaction time for each test was maintained strictly as per manufacturer’s protocol, and rigorously constant for each test. The absorbance was measured with light path of 1 cm. The analytical coefficient of variation for estimation of urea, creatinine and uric acid were 3.12%, 4.45% and 3.38% respectively, and the limits of detection were 1 mg/dL, 0.1 mg/dL and 0.15 mg/dL respectively.

### Renal histology

Mid-longitudinal, 5 µm thick sections of kidney were taken, using cryostat (Thermo Shandon, UK), on poly-L-lysine coated slides, and stained using Haematoxylin-Eosin staining procedure, following Mazumder *et al*.^23^. Photographs were taken under bright-field illumination using digital SLR camera attached with microscope (Eclipse C*i*, Nikon, Japan).

### Brain Histology

Histological study of the different brain regions was performed to elucidate any deposition of crystals of adenine and 2,8-dihydroxyadenine, using Nissl and Picro-Sirius red staining procedures, following protocols as standardized in our laboratory^23^. For Nissl staining, 20 µm thick coronal brain sections passing through the cortex, striatum, hippocampus and substantia nigra regions of brain were taken on poly-L-lysine coated slides, dried, hydrated in decreasing alcohol gradient, stained with 0.5% cresyl violet, washed, dehydrated in increasing ethanol gradient, cleared in xylene, mounted in DPX, and photographed at low (4×) and high (20×) magnification under bright-field illumination. For Picro-Sirius red staining, 5–7 µm sections were taken on coated slides, cleared in xylene, hydrated in decreasing alcoholic grades, stained with Haematoxylin, washed in running water, stained with Picro-Sirius red (1% Sirius red in saturated solution of picric acid), washed with acidified water (0.5% acetic acid), removed water by vigorous shaking, dehydrated in absolute alcohol, cleared in xylene, mounted in DPX and photographed under bright-field illumination, using digital SLR camera attached with microscope (Eclipse C*i*, Nikon, Japan).

### Determination of AChE activity

Since CKD is associated with dementia and cognitive decline^17,21^, it is thought prudent to estimate the activity of AChE in different brain regions, which was done from brain slices (histoenzymological) as well as brain tissue homogenates.

### AChE histoenzymology

AChE activity was estimated from brain tissue sections, following Paul and Borah^32^. Briefly, mice were anaesthetised, and perfused with 30 mL each of ice-cold phosphate buffered saline (PBS, 0.1 M, pH 7.4) and 10% v/v glycerol. Brains were harvested, cryoprotected in 30% w/v sucrose solution overnight, and 20 µm thick sections were taken from the different brain regions – prefrontal cortex, cerebral cortex, striatum, amygdala, hippocampus and substantia nigra, on poly-L-Lysine coated slides using cryostat (Themo Shandon, UK). The sections were incubated in a reaction mixture containing 0.1 M tri-sodium citrate, 60 mM copper sulphate, 30 mM potassium ferricyanide and acetylthiocholine iodide (substrate), in 0.1 M PBS (pH 6.0), in dark at 37 °C. After incubation, the slides were washed in PBS, air dried, mounted in DPX and photographed using digital SLR camera attached with microscope (Eclipse C*i*, Nikon, Japan).

### AChE by Ellman’s method

Following Ellman’s method^36^, with minor modifications^35^, AChE activity was determined from tissue homogenates of different brain regions. The method is based on hydrolysis of acetylthiocholine by AChE to produce thiocholine, which reacts with DTNB to produce a colour complex (TNB), which has peak absorbance at 412 nm. Briefly, mice were sacrificed by cervical dislocation, the brains were dissected out, and the different brain regions (prefrontal cortex, cerebral cortex, striatum, hippocampus, amygdala and substantia nigra) were separated or micropunched. The brain regions were homogenized in Tris-HCl buffer (50 mM; pH 7.4) in 1:20 ratio (w/v). The reaction mixture contains 0.1 mL tissue homogenate and 0.8 mL of DTNB in 0.9 mL of Tris–HCl buffer. The reaction was initiated by addition of 0.2 mL of acetylthiocholine iodide, and the absorbance was recorded for 1 min after the addition of the substrate, at 25 °C using spectrophotometer^32^ (Thermo Fisher Scientific, Finland).

### TH-immunohistochemistry

TH-immunohistochemistry was performed following Paul *et al*.^37^. Briefly, mice were anaesthetised, perfused with 30 mL each of PBS (0.1 M, pH 7.4) and 4% w/v paraformaldehyde. The brains were harvested and stored in the same fixative for 48 hours, and then cryoprotected in 30% w/v sucrose solution, pending immunohistochemistry. Coronal brain sections (35 µm thick) passing through the striatum and substantia nigra were made using cryostat (Themo Shandon, UK), and taken in well-plate. The sections were washed three times in 0.1 M Tris-buffered saline (TBS; pH 7.4) for 5 min each, treated with 3% v/v H_2_O_2_ (in 0.1 M TBS; pH 7.4) for 5 min, permeabilized in 0.3% v/v Triton X-100 (in 0.1 M TBS; pH 7.4) for 30 min, blocked in 10% v/v donkey serum containing 0.3% Triton X-100 (in 0.1 M TBS; pH 7.4) for 1 h, washed in 0.1 M TBS, and incubated overnight at 4 °C in primary antibody (rabbit anti-TH IgG, polyclonal; 1:700) in a mixture of 2% v/v donkey serum and 0.1% v/v Triton X-100. After a gentle wash in 0.1 M TBS, the sections were incubated in HRP-conjugated secondary antibody (goat anti-rabbit IgG; 1:1000) in a mixture of 0.1 M TBS (pH 7.4), 2% v/v donkey serum and 0.1% Triton X-100 for 1 h at room temperature. Following washing of unbound antibody in TBS, the sections were incubated in DAB-liquid substrate system for colour development. The sections were then washed in TBS, dehydrated in increasing ethanol grades, cleared in xylene, mounted in DPX and photographed at 4× magnification under bright-field illumination, using digital SLR camera attached with microscope (Eclipse C*i*, Nikon, Japan). Neurons from every sixth serial section of the substantia nigra were counted (n = 6 per group), while photographs of the sections of the striatum were analysed for densitometry using ImageJ (Fiji version) software.

### Rapid Golgi staining

Rapid Golgi staining was performed following Chakraborty *et al*.^38^ with minor modifications. Briefly, the anaesthetised mice were perfused with 30 mL of 0.1 M PBS (pH 7.4), and then fixed transcardially with 30 mL of Rapid Golgi fixative containing a mixture of 5% chloral hydrate, 5% potassium dichromate, 3.2% formaldehyde, 1.5% gluteraldehyde and 1% DMSO. Brains were immediately harvested and different brain regions were dissected out, and stored in the same fixative in dark at room temperature. The fixative was changed after 24 h and kept undisturbed for 48 h, after which the tissues were repeatedly washed with 0.75% silver nitrate until the colour of potassium dichromate was washed away. The tissues were then impregnated with silver nitrate for another 48 h, dehydrated in ethanol grades (30%, 50% and 70%), and 60 µm thick sections passing through cortex and hippocampus were made using cryostat (Themo Shandon, UK). The sections were collected in 70% ethanol, cleared in xylene and mounted in DPX. Photographs were taken using digital SLR camera attached with microscope (Eclipse C*i*, Nikon, Japan) under bright-field illumination to assess neuronal arborisation and dendritic spine density.

## Determination of the Activity of Mitochondrial Complexes

### Mitochondrial complex-I

Mitochondrial complex-I activity was assayed from the cortex, striatum, hippocampus and substantia nigra of the mice, following Pandey *et al*.^39^. The brain regions were dissected out or micro-punched, and 10% w/v homogenate of the brain tissues were prepared in ice-cold buffer containing 0.32 mol/L sucrose in 10 mmol/L potassium phosphate buffer (pH 7.2). The homogenates were centrifuged at 1000 × g for 10 min at 4 °C, using Refrigerated Centrifuge (Eppendorf, Germany). The supernatant was collected and centrifuged at 10,000 × g for 30 min at 4 °C, and the resulting pellet was re-suspended in ice-cold 50 mmol/L Tris–HCl buffer (pH 7.2), and centrifuged at 10,000 × g for 30 min at 4 °C. The pellets were re-suspended in cold 10 mmol/L potassium phosphate buffer (pH 7.2), and used for the enzymatic assay of the mitochondrial complex-I. The reaction mixture contained 10 mM potassium phosphate (pH 7.2), 5 mM sodium azide, 50 mM coenzyme Q_0_ and 70–120 mg tissue extract (final reaction volume, 1 mL). After pre-incubation for 3 min at 32 °C, the reaction was initiated by the addition of NADH (final concentration 120 mM), and the rate of decrease in the absorbance was monitored at 340 nm for 3 min using spectrophotometer (Thermo Fisher Scientific, Finland). The specific enzyme activity was expressed as nmoles of NADH oxidized/min/mg protein (taking extinction coefficient of NADH as 6.2/mM/cm at 340 nm).

### Mitochondrial complex-II

The activity of the complex was assayed using histoenzymological technique, following Riddle *et al*.^40^ with minor modifications^32^. Briefly, anaesthetised mice were transcardially perfused with 30 mL each of PBS (0.1 M, pH 7.4), and 10% v/v glycerol. Brains were dissected out, cryoprotected in 30% w/v sucrose solution overnight, and 20 µm thick sections through the cortex, striatum, hippocampus and substantia nigra were taken on poly-L-lysine coated slides using cryostat (Themo Shandon, UK). Sections were activated in PBS (0.1 M; pH 7.4) for 10 min at 37 °C, and then incubated in a reaction mixture in dark at 37 °C for 30 min. The reaction mixture contained 0.3 mol/L NBT, 0.05 mol/L phosphate buffer (pH 7.4) and 0.05 mol/L sodium succinate. After incubation, the sections were washed, mounted in glycerol and photographed immediately under bright-field illumination, using digital SLR camera attached with microscope (Eclipse C*i*, Nikon, Japan). The images were subjected to densitometric analysis.

### Mitochondrial complex-III

The activity of the complex was assayed using histoenzymology, following Govindaiah *et al*.^41^ with minor modifications^32^. Briefly, anaesthetised mice were transcardially perfused with 30 mL each of PBS (0.1 M, pH 7.4) and 10% v/v glycerol. Following overnight cryoprotection in 30% w/v sucrose solution, 20 µm thick sections through the cortex, striatum, hippocampus and substantia nigra were taken using cryostat (Themo Shandon, UK) on pre-coated slides. The sections were incubated in a reaction mixture containing 50 mg DAB, 20 mg cytochrome c, 4 g sucrose and 90 mL of 0.1 M phosphate buffer (pH 7.4) for 1 h in dark. The sections were washed in phosphate buffer, mounted in glycerol, coverslipped and photographed immediately under bright-field illumination using digital SLR camera attached with microscope (Eclipse C*i*, Nikon, Japan). The images were subjected to densitometric analysis.

## Estimation of Oxidative Stress in Brain

### Superoxide dismutase (SOD) activity

Cu/Zn-SOD activity was analysed from the cortex, striatum, hippocampus and substantia nigra of control and CKD mice brain, following the method as described earlier^42^. Briefly, the mice were sacrificed by decapitation, and different brain regions were immediately dissected out or micro-punched. A 10% w/v homogenate of the different brain regions was prepared in TNG-T buffer [50 mM Tris-HCl (pH 7.4), 150 mM NaCl, 10% glycerol, 1% Triton X-100 and proteinase inhibitor], and then sonicated with 5 strokes at 40 Hz, using Sonicator (Q-Sonica, USA). The tissue suspension was centrifuged for 10 min at 1000 × g, and the supernatant was used for the estimation of SOD activity. The assay mixture (1 mL) contained 0.2 mM of pyrogallol, 1 mM of EDTA and 50 mM of Tris-HCl buffer (pH 8.2). Auto-oxidation of pyrogallol was measured using spectrophotometer (Thermo Fisher Scientific, Finland) at 420 nm for 3 min, with or without the enzyme. The inhibition of pyrogallol oxidation was linear with the activity of the enzyme present. Fifty percent inhibition in pyrogallol auto-oxidation/min/mg protein was considered as one unit of the enzyme activity.

### Catalase (CAT) activity

The activity of CAT was estimated from the cortex, striatum, hippocampus and substantia nigra of the control and CKD mice brain, following Paul *et al*.^42^. Using the same enzyme extract that was used for determination of the SOD activity, CAT activity was also determined. Based on the estimated level of protein, the supernatant was suitably diluted using 0.01 M PBS (pH 7.0) to give 75–100 µg protein in 0.1 mL of enzyme extract. The reaction mixture contained 1 mL of 0.01 M PBS, 0.4 mL distilled water, and 0.5 mL 0.2 M H_2_O_2_. The reaction was initiated by addition of the enzyme extract in the reaction mixture. Absorbance, at 240 nm, was recorded using spectrophotometer (Thermo Fisher Scientific, Finland) for 5 min whereby H_2_O_2_ is decomposed and the optical density decreased. The enzyme activity was represented as change in absorbance/min/mg protein.

### Lipid peroxidation assay

The level of thiobarbituric acid-reactive substance (TBARS) was determined in the tissue homogenates of different brain regions: cortex, striatum, hippocampus, and substantia nigra, following Koudelová and Mourek^43^. In brief, mice were sacrificed by decapitation, brains were harvested, different brain regions were isolated, and 10% w/v brain tissue homogenate was prepared in PBS (0.1 M, pH 7.4), and incubated at 95 °C for 60 min in acid medium containing 0.45% sodium dodecyl sulphate and 0.6% TBA. After centrifugation, the absorbance of the reaction product (TBARS) was determined at 532 nm using spectrophotometer (Thermo Fisher Scientific, Finland). 1,1,3,3-tetramethoxypropane was used as standard, and the results were expressed as nmol TBARS/mg protein. All the reagents used for the assay were of analytical reagent grade, and the purities were ≥98% for TBA (T5500, Sigma-Aldrich Co., USA), 99% for SDS (54468, SISCO Research Laboratories, India) and 99% for 1,1,3,3-tetramethoxypropane (108383, Sigma Aldrich Co., USA). The analytical coefficient of variation for the estimation of TBARS was 13.28%, and the limit of detection was 0.1 nmol TBARS/mg protein.

### Determination of inflammation in brain

To assess inflammatory stress on neurons, the extent of nitric oxide synthase (nNOS)-active neurons were determined by histoenzymological method. For reactive glia (astroglia), GFAP-immunohistochemistry was performed.

### GFAP-immunohistochemistry

GFAP–immunohistochemistry was performed following Paul *et al*.^37^ from the cortex, striatum, hippocampus and substantia nigra of the brain. The procedure was similar to the one used for TH-immunohistochemistry except that the primary antibody used was rabbit anti-GFAP (1:500), and the sections were photographed at low (4×) and high (20×) magnification under bright field illumination, using digital SLR camera attached with microscope (Eclipse C*i*, Nikon, Japan).

### nNOS histoenzymology

The extent of nNOS-active neurons were assayed from different brain regions using histoenzymological technique, following Hope *et al*.^44^. Briefly, the anaesthetised mice were perfused with 30 mL 10% v/v glycerol, brains were harvested, cryoprotected in 30% w/v sucrose solution overnight, and 20 µm thick coronal brain sections passing through cortex, striatum, hippocampus and substantia nigra were made using cryostat (Themo Shandon, UK). Brain sections were taken on poly L-lysine coated slides, and were permeabilized with 50 mM Tris-HCl buffer (pH 7.4) containing 0.2% Triton X-100. The sections were then incubated in a reaction mixture containing 50 mM Tris-HCl buffer (pH 8.0), 15 mM CaCl_2_, 10 mM NADPH, 5 mM NBT and 0.2% Triton X-100, for 1 hour at 37 °C in dark. Following the development of colour, the slides were washed in Tris-HCl buffer (pH 7.4) twice, mounted in DPX and photographed at low (4×) and high (20×) magnification on the same day, using digital SLR camera attached with microscope (Eclipse C*i*, Nikon, Japan).

### Protein estimation

Protein content in the brain tissue homogenate was determined following Lowry *et al*.^45^ using bovine serum albumin as standard.

### Image analysis

The images of serial sections of TH-immunoreactivity of the striatum, and mitochondrial complex-II and complex-III histoenzymological studies were analysed using ImageJ (Fiji version) software for the determination of optical density^23,46^. Optical density = log (maximum grey value/mean grey value). For counting the number of TH-positive neurons in the substantia nigra, using ImageJ software, every sixth serial section from each mice (n = 6) was selected and the total number of neurons in those sections was counted^37^.

### Statistical analysis

Statistical analysis was performed employing a parametric unpaired Student’s *t*-test (two-tailed) or a two-way, repeated measures ANOVA using GraphPad Prism version 7.0 (GraphPad software, San Diego, CA, USA). Each group consisted of at least six animals. The values are expressed as mean ± S.D., and the distribution of data points have been shown using whisker box plot mean with 95% confidence interval. The analytical coefficient of variation for estimation of haematological parameters and lipid peroxidation were calculated prior to start of the assays to check precision of the assay methods. The difference of mean was considered statistically significant at a P-value of 0.05 or less.

## Results

### High Adenine diet leads to decrease in body weight in mice

#### Decrease in body weight

**Figure 1:**
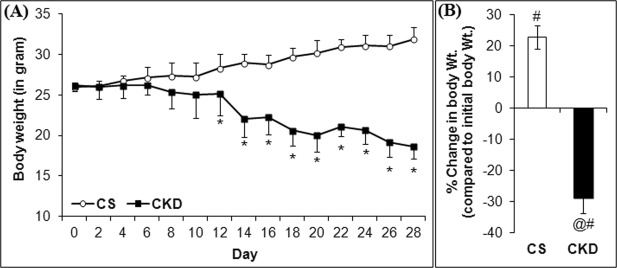
High adenine diet leads to: (**A**) a gradual decline in body weight in mice, and (**B**) a significant decrease in % change in body weight compared to the initial body weight. The control (CS) mice received standard diet, while chronic kidney disease (CKD) group were given adenine at the dose of 0.3% w/w mixed with standard feed for 4-weeks, and body weight was taken every alternative day. *P ≤ 0.05 as compared to CS was considered statistically significant, n = 6. Data were analyzed using a two-way repeated measure ANOVA.

In the present study, the mice given adenine-rich diet had a gradual decrease in the body weight from the 12^th^ day onwards, while in the control group there had been a gradual increase in body weight (Fig. 1A). By the end of the experiment, the mice given high adenine diet had a significant decrease in body weight by 28.89 ± 4.69% compared to the initial body weight, while control mice gained weight by 22.92 ± 3.65% (Fig. 1B).

### High adenine diet leads to behavioural abnormalities in mice

#### Motor behavioural abnormality

**Figure 2:**
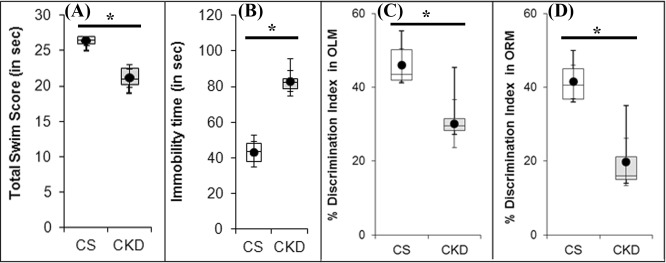
High adenine diet causes psycho-motor behavioural abnormalities, and cognitive decline. The control (CS) mice received standard diet, while chronic kidney disease (CKD) group were given adenine at the dose of 0.3% w/w mixed with standard feed for 4-weeks, and the tests were performed at the end of 4 weeks treatment. Distribution of (**A**) total swim scores, (**B**) total immobility time, (**C**) DI of Short-term Object Location Memory (OLM; 90 min) and (**D**) DI of Long-term Object Recognition Memory (ORM; 24 h), in CS and CKD groups shown as box plot. The box extends from 25^th^ to 75^th^ percentile, the horizontal line inside the box represent median, and the whiskers delimit minimum to maximum values. The dot within the box represents the mean of the group with 95% confidence interval represented as error bars. *P ≤ 0.05 as compared to CS was considered statistically significant, n = 6. The CS mice received standard diet while CKD mice were given adenine at the dose of 0.3% w/w mixed with standard feed for 4-weeks. The results indicate that the CKD group had significant psycho-motor and cognitive deficit. Discrimination index (DI) was calculated as: [(time exploring the novel object − time exploring the familiar)/(time exploring novel + familiar) × 100]. Data were analyzed using an unpaired two-tailed Student’s *t*-test.

The present study demonstrated that the CKD mice developed significant motor behavioural abnormalities as revealed by the decrease in swim scores from 7^th^ minute onwards, and thus the CKD mice had a significantly reduced total swim score (26.33 ± 0.82), compared to the control mice (19.67 ± 2.42) (Fig. 2A; Supplementary Table 1).

#### Psychometric behavioural abnormality

In the forced swim test, the total immobility time of the control mice was 43.67 ± 7.12 sec, while the CKD mice exhibited increased immobility time of 83.17 ± 7.25 sec. Thus, with a significant increase by 1.9-fold in the immobility time in forced swim test, the CKD mice were found to exhibit depression-like behaviour (Fig. 2B; Supplementary Table 1).

#### Cognitive functional deficit

During training sessions of OLM and ORM, there occurred no significant differences in total number and time of exploration in both the objects in mice given high adenine diet, compared to the control. From the time of exploration between the objects in OLM and ORM tests DI was calculated and expressed in percentage. In OLM test, mean percentage of DI in the CKD was found to be 30.17 ± 1.66, while in control it was 46.00 ± 2.91 (Fig. 2C; Supplementary Table 1). The DI was decreased by 16% in OLM test in the CKD mice which differ significantly compared to the control animals. In ORM test, mean percentage of DI in the CKD mice was found to be 19.75 ± 3.30, while in control it was 41.50 ± 2.31 (Fig. 2D; Supplementary Table 1). The DI in ORM test was found to be decreased significantly by 22% in the CKD mice, compared to the control. Thus, the CKD mice exhibited significantly retarded short-term as well as long-term memory compared to the control animals.

Therefore, high adenine diet has been found to cause psycho-motor behavioural abnormalities as well as short-term and long-term cognitive functional deficit in mice.

### High adenine diet leads to CKD in mice

#### Increase in serum urea level

**Figure 3:**
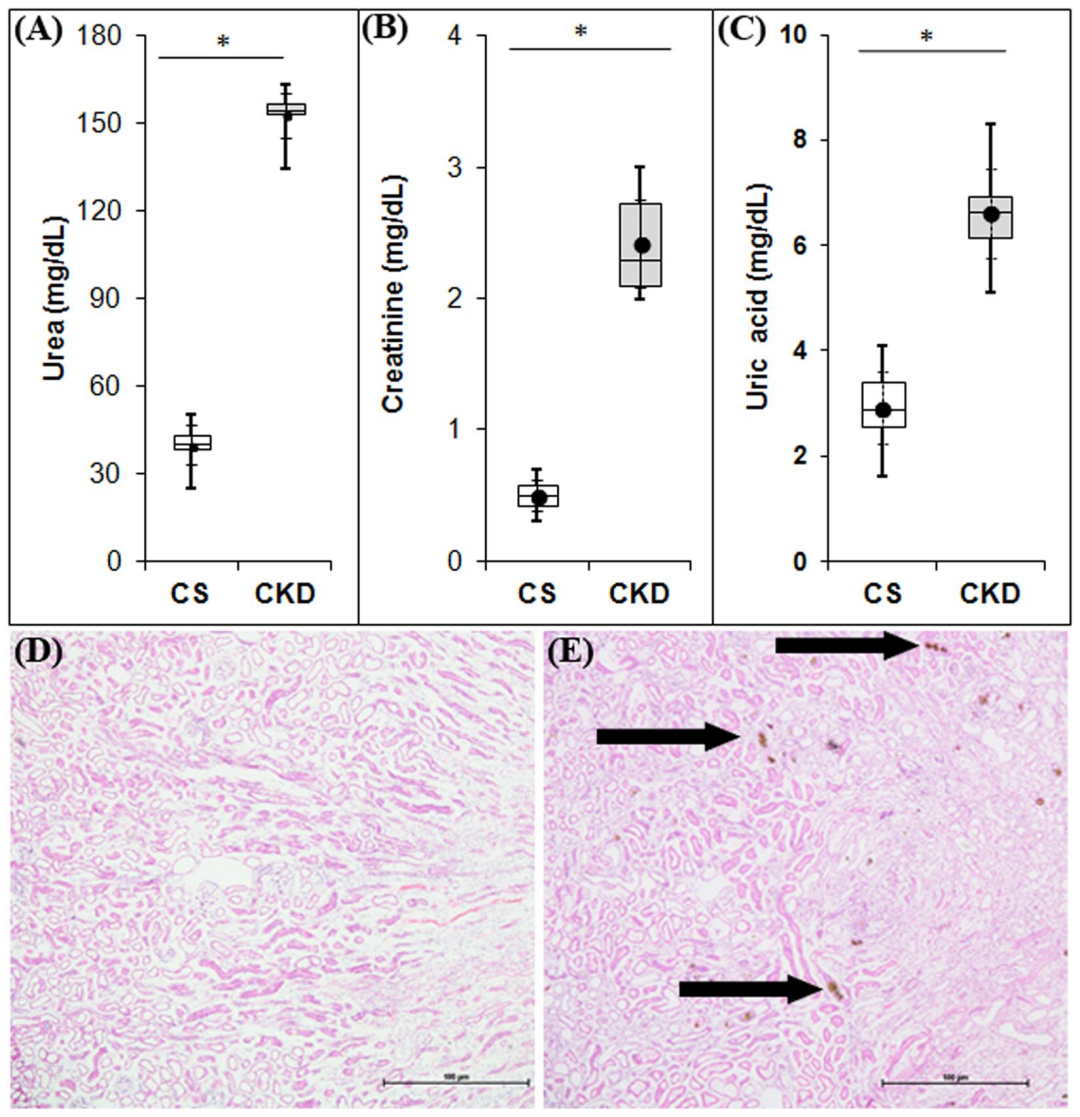
High adenine diet leads to chronic kidney disease (CKD) in mice with elevation in the level of serum urea (**A**), creatinine (**B**) and uric acid (**C**), and deposition of adenine and 2,8-dihydroxyadenine crystals in the renal tissues of mice (**E**). Distribution of urea (**A**), creatinine (**B**) and uric acid (**C**) values in different groups: control (CS) and CKD. The CS mice received standard diet while CKD mice were given adenine at the dose of 0.3% w/w mixed with standard feed for 4-weeks. Serum levels of urea, creatinine and uric acid were tested at the end of the experiment, using commercially available kits. The boxes (in **A**, **B** and **C**) extends from 25^th^ to 75^th^ percentile, the horizontal lines inside the boxes represent median, and the whiskers delimit minimum to maximum values. The dots within the boxes represents the mean of the group with 95% confidence interval represented as error bars. Data were analyzed using an unpaired two-tailed Student’s *t*-test and *P ≤ 0.05 as compared to CS was considered statistically significant, n = 6. (**E**) Renal histology showing adenine and 2,8-dihydroxyadenine crystals in the renal tissues in both cortical and medullary regions of CKD mice (arrowheads) as uroliths, while no such deposits were found in CS mice (**D**). Photographs were taken under bright field illumination at 4× magnification.

The serum urea levels have been found to be 39.72 ± 8.50 mg/dL and 152.90 ± 9.64 mg/dL in the control group and the mice given adenine-rich diet respectively (Fig. 3A; Supplementary Table 1). Thus, there was a significant increase in the serum urea level by 3.85–fold in the mice fed with adenine-rich diet.

#### Increase in serum creatinine

The serum creatinine levels were found to be 0.50 ± 0.14 mg/dL and 2.42 ± 0.42 mg/dL respectively in the control group and the mice given adenine-rich diet (Fig. 3B; Supplementary Table 1), thereby showing a significant increase in the serum creatinine level by 4.83-fold in the adenine-fed mice.

#### Increase in serum uric acid

The serum uric acid level in the mice given high adenine diet was found to be 6.62 ± 1.06 mg/dL, while in the control group the level was 2.92 ± 0.86 mg/dL (Fig. 3C; Supplementary Table 1). Thus, there was a significant increase in serum uric acid level by 2.27-fold in the mice given adenine-rich diet.

#### Renal histological damages

Histological studies using Hematoxylin-Eosin staining revealed that adenine feeding caused deposition of adenine and 2,8-dihydroxyadenine crystals in the renal tissues as uroliths, both in the cortex and medulla regions (Fig. 3E). Moreover, leukocyte infiltration and foreign body granulomas were also seen in the renal sections of the mice given adenine-rich diet (Fig. 3E). There were no such pathologies in the renal tissue sections of the control mice (Fig. 3D).

Thus, high adenine diet, at the dose of 0.3% w/w mixed with feed for 4 weeks, leads to haematological and histopathological features of chronic kidney disease in mice.

#### Brain histology

**Figure 4:**
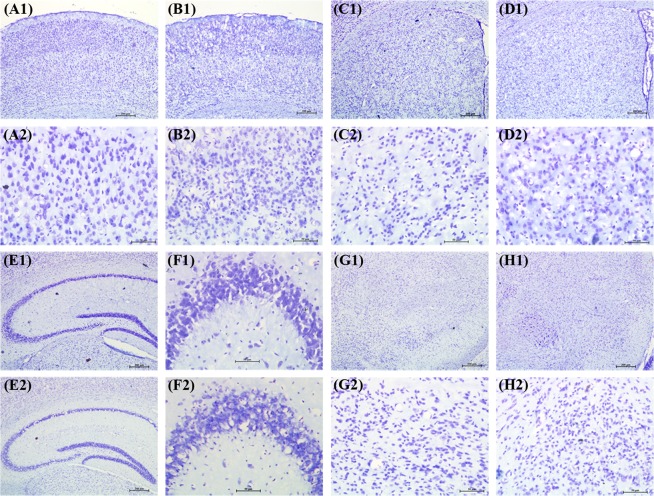
Representative sections of the Nissl staining of different brain regions of control (CS) and chronic kidney disease (CKD) mice. 20 µm thick coronal brain sections passing through cortex (**A1**,**A2**,**B1**,**B2**), striatum (**C1**,**C2**,**D1**,**D2**), hippocampus (**E1**,**E2**,**F1**,**F2**), and substantia nigra (**G1**,**G2**,**H1**,**H2**) were stained with cresyl violet. Neither morphological aberrations nor depositions of the crystals of adenine and 2,8-dihydroxyadenine were found in the brain regions of control (**A1**,**A2**,**C1**,**C2**,**E1**,**F1**,**G1**,**G2**) or CKD mice (**B1**,**B2**,**D1**,**D2**,**E2**,**F2**,**H1**,**H2**). The different brain regions were photographed at low, 4× (**A1**,**B1**,**C1**,**D1**,**E1**,**E2**,**G1**,**H1**), and high, 20× (**A2**,**B2**,**C2**,**D2**,**F1**,**F2**,**G2**,**H2**) magnification under bright field illumination. F1 and F2 are the Cornus Ammonis 3 (CA3) regions of the Hippocampus of E1 and E2, of control and CKD mice respectively.

**Figure 5:**
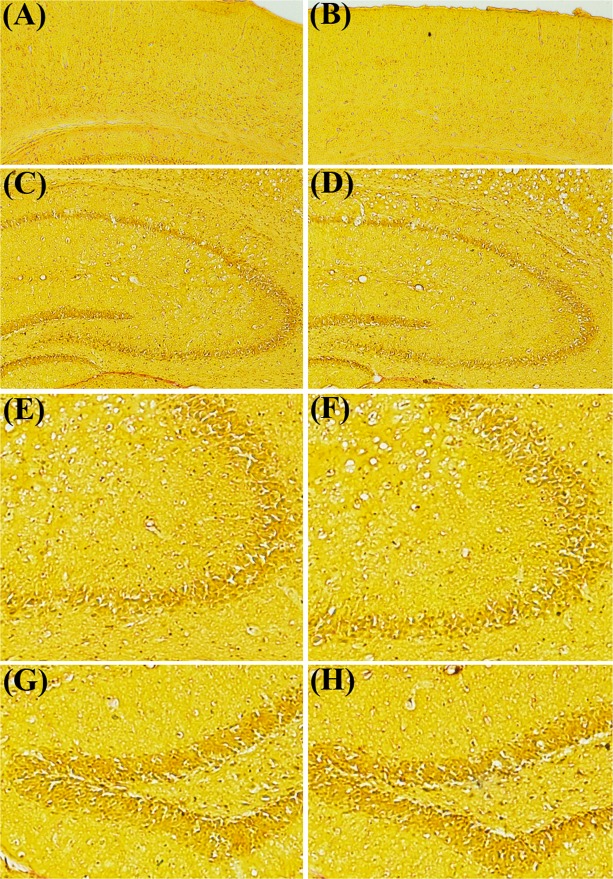
Representative photographs of the different brain regions: Cortex (**A**,**B**), Hippocampus (**C,D**), Hippocampal Cornus Ammonis 3 (**E**,**F**), and Hippocampal Dentate Gyrus (**G**,**H**), of control (**A**,**C**,**E**,**G**) and chronic kidney disease (**B**,**D**,**F**,**H**) mice stained with Picro-Sirius red. 5–7 µm thick coronal brain sections were processed for Picro-Sirius red staining. The photographs were taken at low, 4× (**A**–**D**), and high, 10× (**E**–**H**) magnification under bright field illumination.

Neither Nissl staining (Fig. 4) nor Picro-Sirius red staining (Fig. 5) revealed any deposition of crystals of adenine and 2,8-dihydroxyadenine in any of the studied brain region of the CKD mice.

#### CKD in mice leads to decrease in brain AChE activity

**Figure 6:**
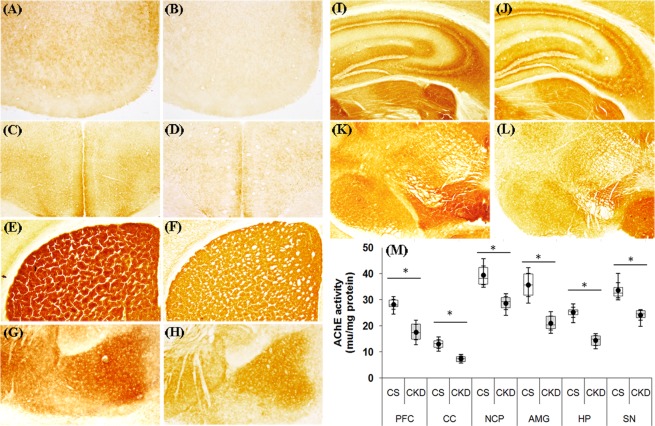
Acetylcholinesterase (AChE) activity in different brain regions of control (CS) and chronic kidney disease (CKD) mice. Representative sections of AChE histoenzymology in different brain regions: (**A,B**) prefrontal cortex (PFC), (**C,D**) cerebral cortex (CC), (**E,F**) striatum (NCP), (**G,H**) amygdala (AMG), (**I,J**) hippocampus (HP) and (**K,L**) substantia nigra (SN). 20 µm thick sections from glycerol perfused brain were stained for AChE histoenzymology. The activity of the enzyme AChE, indicated by colour intensity, was found to be decreased in all the brain regions of CKD mice (**B,D,F,H,J,L**), compared to CS (**A,C,E,G,I,K**). Photographs were taken under bright field illumination at 4× magnification. The activity of AChE (in mu/mg protein) in the brain tissue homogenates from the same regions was also estimated following Ellman method. (**M**) Distribution of AChE activity values in different groups – CS and CKD shown as box plot. The box extends from 25^th^ to 75^th^ percentile, the horizontal line inside the box represent median, and the whiskers delimit minimum to maximum values. The dot within the box represents the mean of the group with 95% confidence interval denoted by error bars. An unpaired Student’s *t*-test (two-tailed) was used to calculate P-values.*P ≤ 0.05 as compared to CS was considered statistically significant (n = 6).

Histoenzymological analysis for the activity of the enzyme Acetylcholinesterase (AChE) revealed a marked visible decrease in the colour intensity, in different brain regions, viz. prefrontal cortex, cerebral cortex, striatum, amygdala, hippocampus and substantia nigra of CKD mice, compared to the control (Fig. 6A–L). Analysis from the brain tissue homogenates of these respective regions, following Ellman’s method, the activity of AChE (in mu/mg protein) was found to be 28.23 ± 2.41, 13.01 ± 1.99, 39.34 ± 4.42, 35.67 ± 5.55, 25.17 ± 2.42, 33.61 ± 3.69 in prefrontal cortex, cerebral cortex, striatum, amygdala, hippocampus and substantia nigra respectively in control mice, while in CKD mice the same was 17.57 ± 3.76, 7.29 ± 1.27, 28.69 ± 3.10, 21.01 ± 3.28, 14.37 ± 2.35 and 24.01 ± 2.45 respectively (Fig. 6M; Supplementary Table 2). Thus, there was a significant decrease in the enzyme activity in prefrontal cortex, cerebral cortex, striatum, hippocampus, amygdala and substantia nigra of CKD mice by 37.76%, 43.97%, 27.07%, 41.09%, 42.95% and 28.56% respectively, compared to control.

#### TH-immunoreactivity in mice with CKD

**Figure 7:**
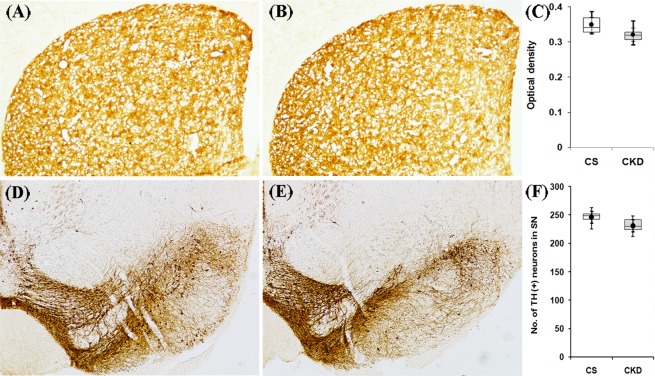
Dopaminergic neuronal status in the nigrostriatum of control (CS) and chronic kidney disease (CKD) mice. Representative sections of tyrosine hydroxylase (TH)–immunohistochemistry in the striatum (**A,B**) and substantia nigra (**D,E**) of CS (**A,D**) and CKD (**B,E**) mice. Photographs were taken at 4× magnification under bright field illumination. Distribution of optical density values in the striatum (**C**) and TH-positive neuronal count in the SN (**F**) of CS and CKD is shown as box plot. The box extends from 25^th^ to 75^th^ percentile, the horizontal line inside the box represent median, and the whiskers delimit minimum to maximum values. The dot within the box represents the mean of the group with 95% confidence interval represented by error bars. Densitometric analysis of the images of striatum didn’t reveal any significant change in the optical density, while every sixth serial section through the SN was taken (n = 6) and the total number of neurons present in those sections were counted, which also revealed no significant alteration in the CKD mice compared to CS. Data were analyzed using a two-way repeated measure ANOVA.

Immunohistochemistry for TH was performed to investigate dopaminergic neuronal status in the striatum and substantia nigra (Fig. 7). Densitometric analysis of the images of striatum revealed that in control mice, the OD was 0.349 ± 0.027, while in CKD mice it was 0.321 ± 0.024 (Fig. 7C; Supplementary Table 2). In the substantia nigra, the numbers of TH-positive neurons were found to be 246.33 ± 12.71 and 231.33 ± 13.43 in control and CKD mice respectively (Fig. 7F; Supplementary Table 2). However, there was no statistically significant change in the dopaminergic neuronal status in the substantia nigra or their axon terminals in the striatum of the CKD mice, compared to the control.

#### CKD in mice leads to dendritic spine loss and decrease in arborisation

**Figure 8:**
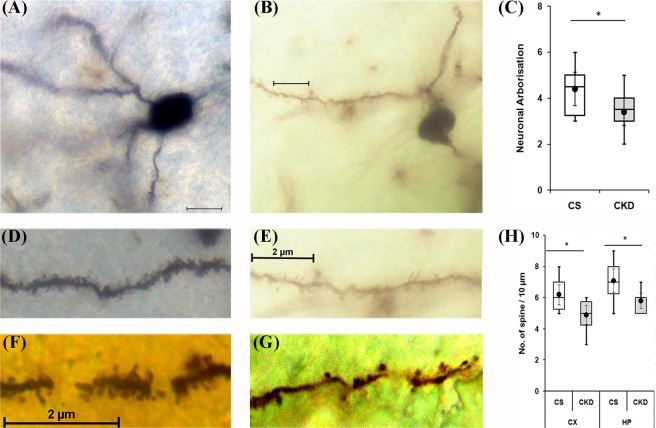
Dendritic spine density and neuronal arborisation in control (CS) and chronic kidney disease (CKD) mice. Representative photographs of Rapid Golgi stained brain sections of CS (**A,D,F**) and CKD (**B,E,G**) mice. A decrease in neuronal arborisation in the (**B**) hippocampus (HP), and dendritic spine density in the (**E**) cortex (CX) and (**G**) HP of CKD mice, compared to CS, was visible. Box plot showing (**C)** distribution of neuronal arborisation in HP, and (**H**) number of spine per 10 µm length of the dendrite in CX and HP, of CS and CKD mice. The box extends from 25^th^ to 75^th^ percentile, the horizontal line inside the box represent median, and the whiskers delimit minimum to maximum values. The dot within the box represents the mean of the group with 95% confidence interval represented by error bars. Scale bar is 2 µm. Photographs were taken at 100× magnification under bright field illumination. Data were analyzed using a two-way repeated measure ANOVA. *P ≤ 0.05 as compared to CS; n = 6.

Rapid Golgi staining was performed in the cortex and hippocampus to reveal neuronal architecture in terms of dendritic spine density and neuronal arborisation. While the neuronal arborisation was found to be decreased only in hippocampus of CKD mice brain (Fig. 8B), compared to control (Fig. 8A), there was a significant decrease in the spine density in the cortex (Fig. 8E) and hippocampus of CKD mice (Fig. 8G), compared to control (Fig. 8D,F; Supplementary Table 2). The hippocampal neuronal arborisation was found to be 4.4 ± 1.07 and 3.4 ± 0.57 respectively in control and CKD mice (Fig. 8C; Supplementary Table 2). Thus, a significant decrease in arborisation by 23% in the hippocampus was evident in CKD mice, compared to control. The spine density in the cortex of the control and CKD mice was found to be 6.2 ± 1.03 and 4.9 ± 0.99 per 10 µm length respectively, while the density in the hippocampus of the control and CKD mice was 7.1 ± 1.19 and 5.8 ± 0.79 respectively (Fig. 8H). Thus, there was a significant decrease in the spine density in the cortex and hippocampus by 21% and 18% respectively.

### CKD in mice leads to dysfunction of mitochondrial complexes

#### Mitochondrial complex-I

**Figure 9:**
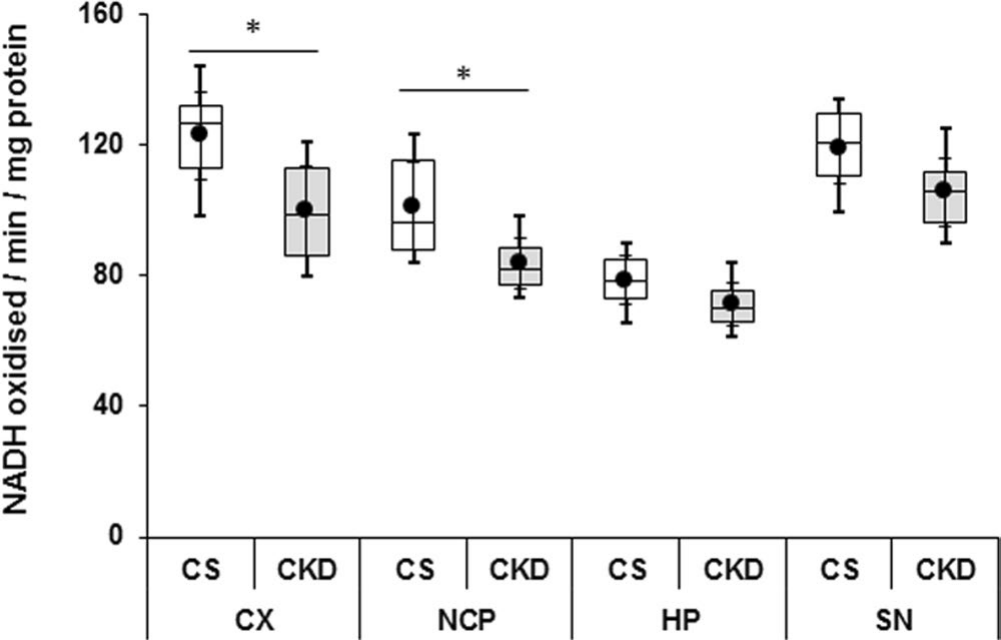
Mitochondrial complex-I activity in different brain regions of control (CS) and chronic kidney disease (CKD) mice: cortex (CX), striatum (NCP), hippocampus (HP) and substantia nigra (SN). Following 4 weeks of high adenine diet in CKD group, mice were sacrificed, brain regions separated, and the enzyme activity was analysed. The activity of the complex is expressed as NADH oxidised/min/mg protein. The box plot shows the distribution of complex-I activity values in different groups, CS and CKD, at different brain regions. The box extends from 25^th^ to 75^th^ percentile, the horizontal line inside the box represent median, and the whiskers delimit minimum to maximum values. The dot within the box represents the mean of the group with 95% confidence interval shown as error bars. Data were analyzed using an unpaired two-tailed Student’s *t*-test. *P ≤ 0.05 compared to CS, n = 6.

The activity of complex-I (expressed as the amount of NADH oxidized/min/mg protein) was found to be 123.12 ± 16.81, 101.34 ± 11.95, 78.67 ± 9.17 and 119.31 ± 13.39 in cortex, striatum, hippocampus and substantia nigra of control mice, while in CKD mice the activities were 99.78 ± 17.08, 83.78 ± 9.49, 71.34 ± 8.20 and 105.78 ± 12.99 respectively (Fig. 9; Supplementary Table 2). However, the decrease in the activity of the complex was found to be significant only in the cortex and striatum of the CKD mice by 18.96% and 17.33% respectively, compared to the control.

#### Mitochondrial complex-II

**Figure 10:**
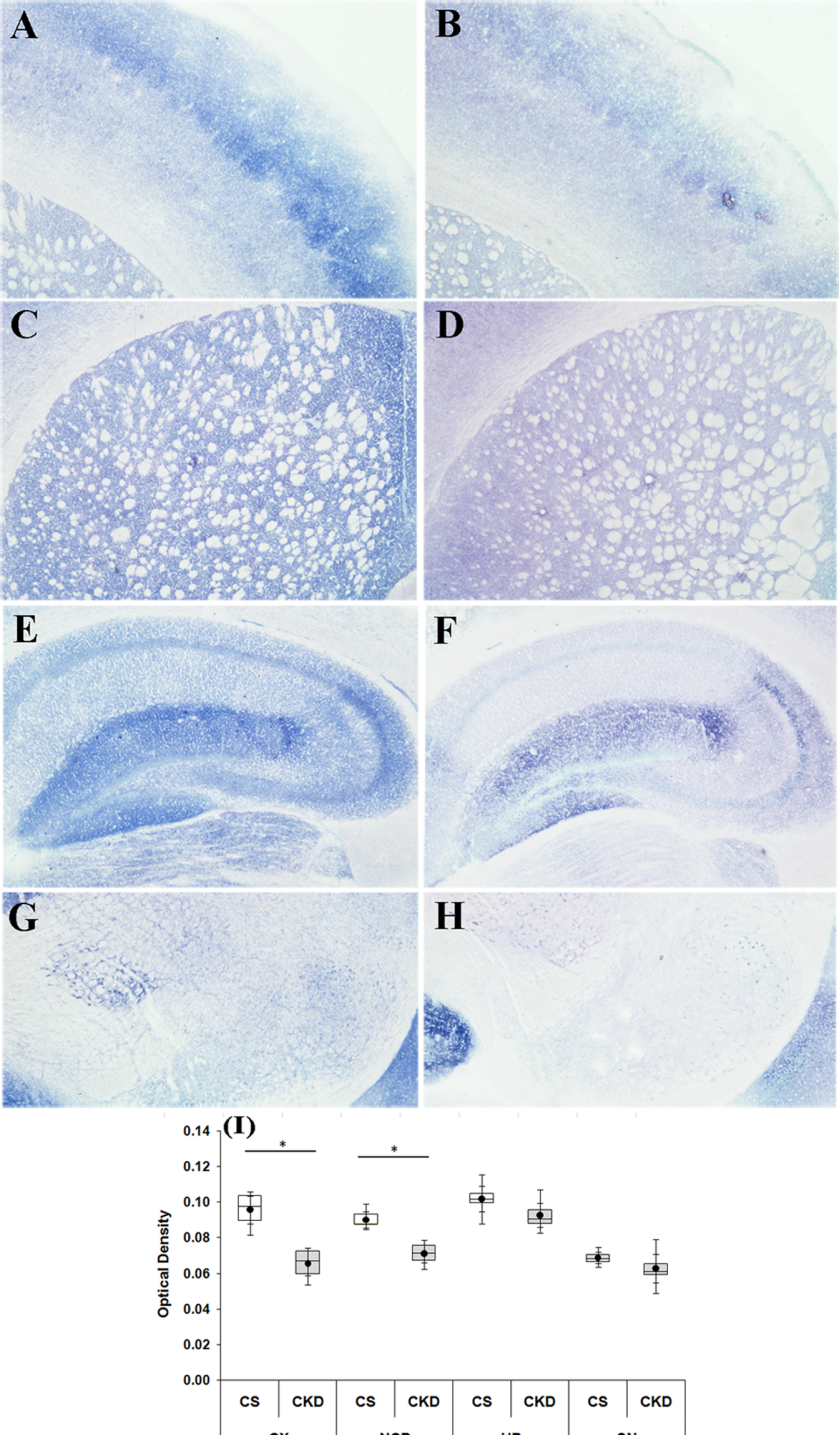
Mitochondrial complex-II activity in different brain regions of control (CS) and chronic kidney disease (CKD) mice. Representative sections of different brain regions of CS (**A,C,E,G**) and CKD (**B,D,F,H**) mice, assayed for mitochondrial complex-II histoenzymology: cortex (CX; **A,B**), striatum (NCP; **C,D**), hippocampus (HP; **E,F**) and substantia nigra (SN; **G,H**). There was a visible decrease in the colour intensity, which signifies decreased activity of the complex. Photographs were taken under bright field illumination at 4× magnification. Densitometric analysis of the images of different brain regions, for determining optical density (OD), revealed a decrease in the activity of the complex in the CX and NCP of CKD mice, compared to CS, while the decrease in HP and SN were insignificant (I). (**I**) Box plot showing distribution of optical density values of different groups in different brain regions. The box extends from 25^th^ to 75^th^ percentile, the horizontal line inside the box represent median, and the whiskers delimit minimum to maximum values. The dot within the box represents the mean of the group with 95% confidence interval shown as error bars. Data were analyzed using a two-way repeated measure ANOVA. *P ≤ 0.05 compared to CS, n = 6.

There was an apparent decrease in the activity of the complex, as revealed by the decrease in the colour intensity in different brain regions of CKD mice, compared to the control (Fig. 10A–H). The photographs were subjected to densitometric analysis for determination of OD. The OD in the cortex, striatum, hippocampus and substantia nigra of the control mice was found to be 0.096 ± 0.010, 0.090 ± 0.006, 0.102 ± 0.009 and 0.069 ± 0.004 respectively, while in CKD mice the same was 0.066 ± 0.009, 0.071 ± 0.006, 0.093 ± 0.009 and 0.063 ± 0.010 in the respective brain regions (Fig. 10I; Supplementary Table 2). However, the decrease in the activity of the complex-II was found to be statistically significant in the cortex and striatum of the CKD mice by 31.58% and 21.18% respectively, compared to the control.

#### Mitochondrial complex-III

**Figure 11:**
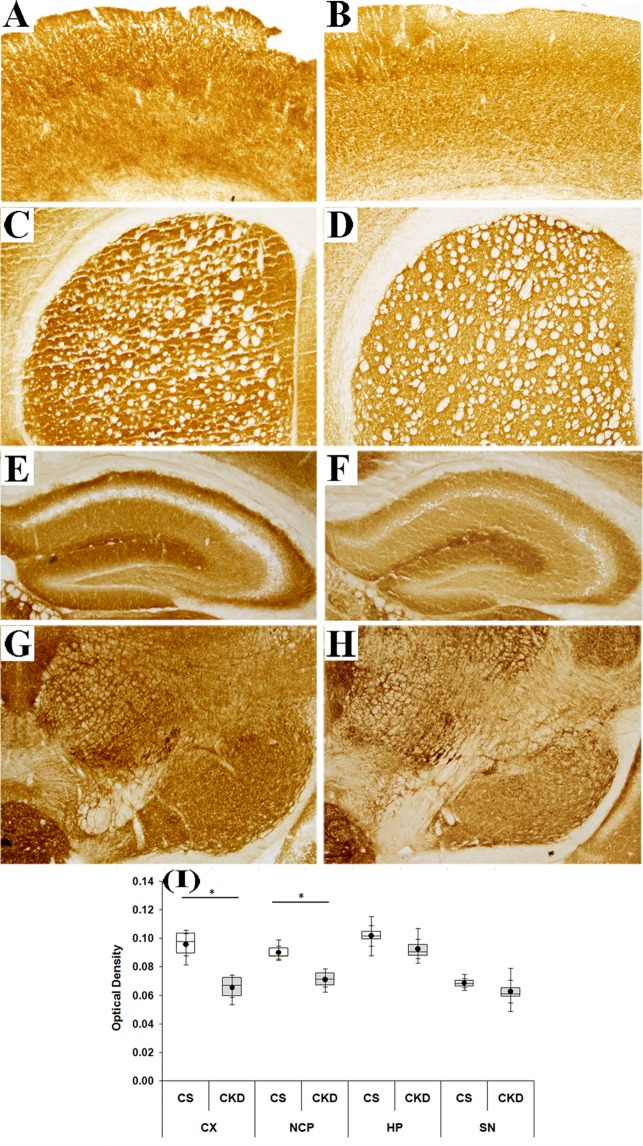
Mitochondrial complex-III activity in different brain regions of control (CS) and chronic kidney disease (CKD) mice. Representative sections of different brain regions of CS (**A,C,E,G**) and CKD (**B,D,F,H**) mice, assayed for mitochondrial complex-III histoenzymology: cortex (CX: **A,B**), striatum (NCP: **C,D**), hippocampus (HP: **E,F**) and substantia nigra (SN: **G,H**). There was a visible decrease in the staining intensity, which signifies decreased activity of the complex in the CKD mice, compared to CS. Photographs were taken under bright field illumination at 4× magnification. Densitometric analysis of the images of different brain regions, for determining optical density (OD), revealed a decrease in the activity of the complex in the CX and NCP of CKD mice, compared to CS, while the decrease in HP and SN were insignificant (I). (**I**) Box plot showing distribution of optical density values of different groups in different brain regions. The box extends from 25^th^ to 75^th^ percentile, the horizontal line inside the box represent median, and the whiskers delimit minimum to maximum values. The dot within the box represents the mean of the group with 95% confidence interval shown as error bars. Data were analyzed using a two-way repeated measure ANOVA. *P ≤ 0.05 compared to CS, n = 6.

There was an apparent decrease in the activity of the mitochondrial complex-III in different brain regions of the CKD mice, compared to control (Fig. 11A–H), as evident by the decrease in colour intensity. Thus, the images were subjected to densitometric analysis. The OD in cortex, striatum, hippocampus and substantia nigra of control mice was found to be 0.226 ± 0.036, 0.228 ± 0.024, 0.246 ± 0.026 and 0.301 ± 0.024 respectively, while in CKD mice the same was 0.183 ± 0.024, 0.188 ± 0.028, 0.229 ± 0.026 and 0.290 ± 0.023 in the respective brain regions (Fig. 11I; Supplementary Table 2). However, the decrease in the OD was found to be statistically significant in the cortex and striatum of the CKD mice by 31.57% and 18.02% respectively, compared to the control.

### CKD in mice leads to oxidative stress in brain

#### Increase in SOD activity

**Figure 12:**
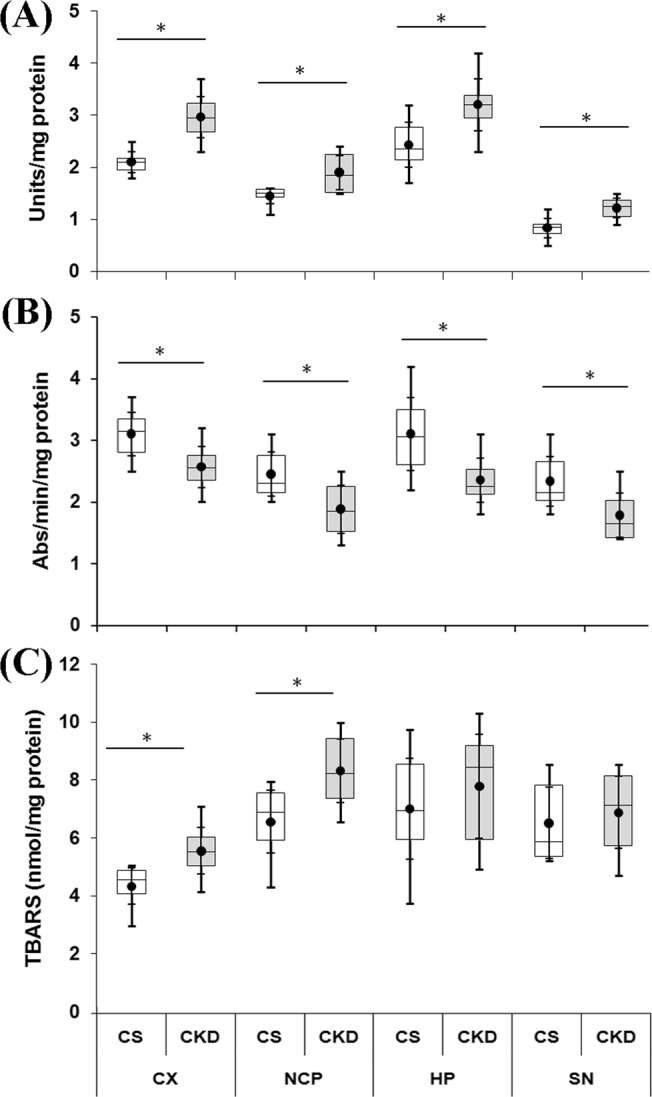
Chronic kidney disease (CKD) leads to oxidative stress in brain. Activity of (**A**) Superoxide dismutase (SOD) and (**B**) Catalase (CAT), and (**C**) level of lipid peroxidation. Mice from control (CS) and CKD group were sacrificed at the end of the treatment period, and different brain regions: cortex (CX), striatum (NCP), hippocampus (HP) and substantia nigra (SN) were isolated and the parameters were tested using standard protocols. SOD and CAT activities were measured as Units/mg protein and Absorbance/min/mg protein respectively, and level of lipid peroxidation was measured as amount of thiobarbituric acid-reactive substance (TBARS) in nmol/mg protein. The box plots show the distribution of activities of SOD and CAT, and the level of lipid peroxidation values of different groups in different brain regions. The box extends from 25^th^ to 75^th^ percentile, the horizontal line inside the box represent median, and the whiskers delimit minimum to maximum values. The dot within the box represents the mean of the group with 95% confidence interval shown as error bars. An unpaired Student’s *t*-test (two-tailed) was used to calculate P-values. *P ≤ 0.05 as compared to CS, n = 6.

SOD is an important marker of oxidative stress. In control mice, SOD activity (expressed as Units/mg protein) was found to be 2.11 ± 0.24, 1.45 ± 0.19, 2.43 ± 0.54 and 0.83 ± 0.23 in the cortex, striatum, hippocampus and substantia nigra respectively. In these brain regions of the CKD mice, the activity of SOD was 2.97 ± 0.50, 1.89 ± 0.41, 3.21 ± 0.63 and 1.22 ± 0.23 respectively (Fig. 12A; Supplementary Table 2). Thus, there was a significant increase in the activity of the enzyme by 1.41-, 1.3-, 1.32- and 1.46-fold in cortex, striatum, hippocampus and substantia nigra respectively of the CKD mice, compared to the control group.

#### Decrease in Catalase activity

Catalase activity (in Abs/min/mg protein) was found to be 33.11 ± 0.24, 2.45 ± 0.15, 3.11 ± 0.53 and 2.33 ± 0.20 in the cortex, striatum, hippocampus and substantia nigra of control mice brain, while the same was 2.57 ± 0.41, 1.89 ± 0.38, 2.35 ± 0.25 and 1.78 ± 0.34 respectively in these brain regions of the CKD mice (Fig. 12B; Supplementary Table 2). Thus, there was a significant decrease in the activity of the enzyme by 17.20%, 23.13%, 24.19% and 23.57% in the cortex, striatum, hippocampus and substantia nigra of the CKD mice respectively, compared to the control group.

#### Elevation in lipid peroxidation

The level of thiobarbituric acid reactive substance corresponds to the level of malondialdehyde (MDA), and therefore the extent of lipid peroxidation, which indicates oxidative stress. The level of MDA (in nmol/mg protein) in the different brain regions of the control mice was found to be 4.35 ± 0.62, 6.57 ± 1.09, 7.01 ± 1.75 and 6.53 ± 1.23 in the cortex, striatum, hippocampus and substantia nigra respectively. In these respective brain regions of the CKD mice, the level of MDA was 55.56 ± 0.81, 8.32 ± 0.58, 7.78 ± 1.80 and 6.89 ± 1.25 respectively (Fig. 12C; Supplementary Table 2), with significant increase by 1.3-fold and 1.27-fold respectively in the cortex and striatum.

### CKD in mice leads to inflammation in brain

#### Glia activation

**Figure 13:**
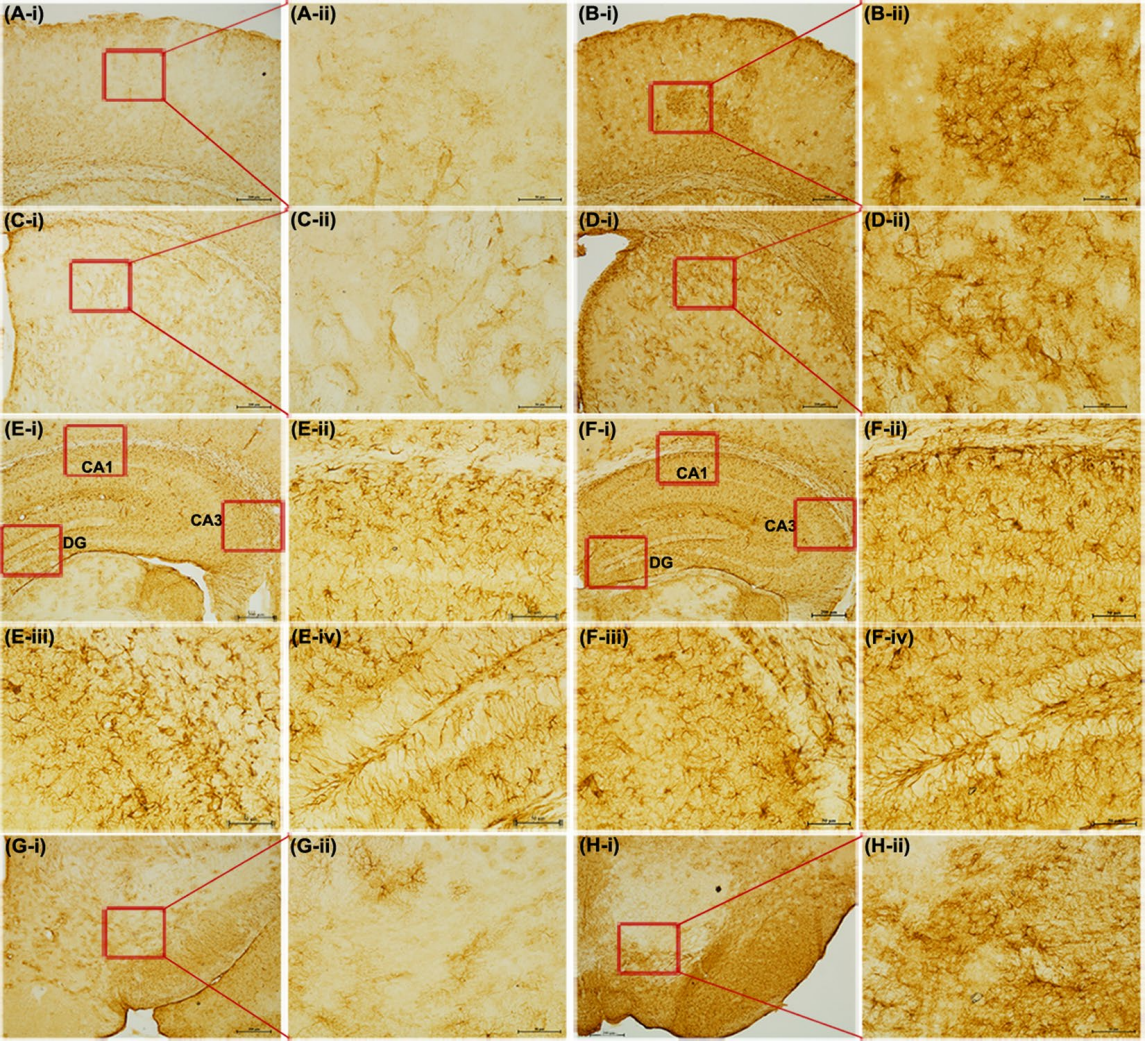
Chronic kidney disease (CKD) leads to Astrocytosis in mice brain. Representative photographs of Glial fibrillary acidic protein (GFAP)-immunohistochemistry in the brain of control (**A,C,E,G**) and CKD (**B,D,F,H**) mice. Note the increased GFAP-reactivity in the cortex (**B-i,ii**), striatum (**D-i,ii**), hippocampal CA1 (**F-ii**) hippocampal CA3 (**F-iii**), hippocampal DG (**F-iv**) and substantia nigra (**H-i,ii**) regions in CKD mice, compared to the corresponding regions of control mice. The photographs A-i, B-i, C-i, D-i, E-i, F-i, G-i, and H-i were taken at 4× magnification, while the remaining photographs A-ii, B-ii, C-ii, D-ii, E-ii-iv, F-ii-iv, G-ii, and H-ii were taken at 20× magnification under bright field illumination. CA = Cornus ammonis; DG = Dentate Gyrus.

GFAP is a marker of the activated astrocytes, and indicates underlying inflammation. The extent of GFAP-positive astrocytes was found to be perceptibly higher in the cortex, striatum, substantia nigra, and different regions of the hippocampus (Cornus Ammonis 1, Cornus Ammonis 3, and Dentate Gyrus) of the CKD mice, compared to the control (Fig. 13), which indicated increase in reactive glial cells in the brain of CKD mice.

#### Activation of nNOS

**Figure 14:**
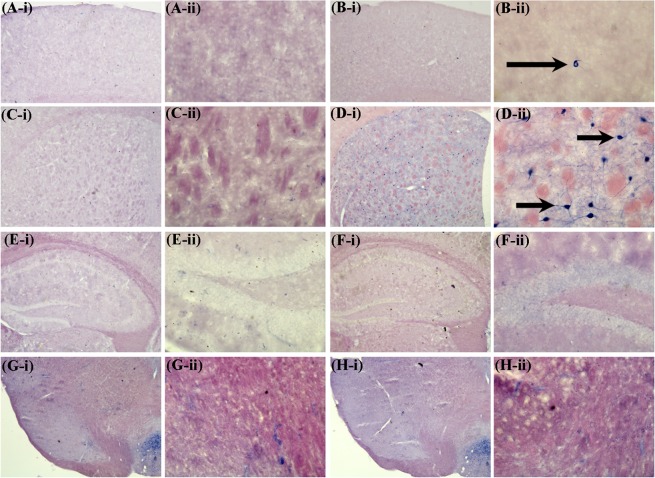
Chronic kidney disease (CKD) in mice leads to activation of neuronal Nitric oxide synthase (nNOS) in brain. Representative sections of nNOS histoenzymology in different brain regions of control (**A,C,E,G**) and CKD (**B,D,F,H**) mice: cortex (**A,B**), striatum (**C,D**), hippocampus (**E,F**) and substantia nigra (**G,H**). Following 4 weeks of high adenine diet in CKD group, mice were sacrificed, glycerol perfused and 20 µm thick coronal brain sections were tested for nNOS histoenzymology. The arrow heads point to the neurons that have active NOS. There are high number of NOS-active neurons in the cortex (**B-ii**) and striatum (**D-ii**) of CKD mice, compared to control. The photographs A-i, B-i, C-i, D-i, E-i, F-i, G-i, and H-i were taken at 4× magnification under bright field illumination, while the others are the magnified (20×) images of the same region.

Activity of nNOS was assayed by histoenzymology from the cortex, striatum, hippocampus and substantia nigra of brain to elucidate the number of NOS-active neurons. In the cortex and striatum of the CKD mice, the NOS-active neurons were extensively seen. However, there was no visible increase in the number of NOS-active neurons in the other brain regions (Fig. 14).

## Discussion

CKD patients suffer from several neurological complications since the disease affects all levels of the nervous system^16–18^. Thus, it is imperative to venture into the mechanisms underlying the neurological complications of CKD, for proper disease management. The present study focuses on understanding the pathophysiology of the neurological complications prevalent in CKD, using mice as model. The mice model of CKD was developed by administration of adenine at the dose of 0.3% w/w mixed with powdered rodent feed for 4 weeks, as per the protocol standardised in our laboratory^23^. The mice given adenine-rich diet had a gradual decline in body weight (Fig. 1), with elevated serum levels of urea (Fig. 3A), creatinine (Fig. 3B) and uric acid (Fig. 3C), which are considered as parameters to ascertain induction of CKD. While elevations in creatinine, urea and uric levels in serum are among conventional serum biomarkers of CKD in human subjects^47,48^, the same have also been consistently reported in rodent models of adenine-induced CKD^23,49–62^. Histological studies revealed extensive deposition of uroliths of adenine and 2,8-dihydroxyadenine, as well as leukocyte infiltration and foreign body granulomas in the renal tissues of the CKD mice (Fig. 3E). These findings are consistent with others^49–62^ and our previous work^23^. In addition, the adenine-induced CKD in rodent models have been reported to cause cardiovascular damages^58^, including increase in left ventricular fibrosis, ventricular stiffness, and wall thickness^56^, increased blood pressure^56,62^, oxidative stress and inflammation including elevation in the activities of SOD, leukocyte infiltration, increased expression of NF-κB^60^, heme oxygenase-1, TNF-α and NOD-like receptor protein-3^56,60^, elevated levels of parathyroid hormone^60,63^, phosphate^56,60^, and anaemia with decrease in erythrocyte count, hematocrit, haemoglobin and erythropoietin^59^, and decrease in food intake^60^, similar to human subjects. This makes the adenine model to be a valid and highly reproducible model, and thereby justifies the use of the model for the present study.

It has been suggested that disturbance in the dopaminergic neurotransmission leads to the motor abnormalities in CKD patients^28–30^, and thus levodopa therapy is practised for amelioration of the same^28,30^. In the present study, the CKD mice exhibited motor behavioural abnormalities (Fig. 2A), which is consistent with previous reports^23,52^. Thus, status of the dopaminergic neurons in the substantia nigra and their axon terminals in the striatum of the brain were assayed, using TH-immunoreactivity. The results revealed no change in the dopaminergic neurons in the nigrostriatum of the brain of CKD mice (Fig. 7). However, in the striatum of the CKD mice, alterations in the activities of SOD (Fig. 12A) and Catalase (Fig. 12B), elevated lipid peroxidation (Fig. 12C), astrocytosis (Fig. 13), increase in NOS-active neurons (Fig. 14) and dysfunction of mitochondrial complexes (Figs 9, 10 and 11) were prevalent; while alterations in SOD (Fig. 12A) and Catalase (Fig. 12B) activities, and astrocytosis (Fig. 13) have been observed in the substantia nigra of the mice. Alterations in the activity of SOD and Catalase, and elevation in lipid peroxidation indicate underlying oxidative stress^64,65^. Activation of nNOS leads to increased production of NO and reactive oxygen and nitrogen species, as well as inflammation^66^, while astrocytosis indicates reactive astroglial cells^35,67^. Further, oxidative stress, inflammation and mitochondrial dysfunctions in brain are among foremost contributors to several neurological diseases^64–72^. Thus, it is hypothesized that these factors might hamper the synthesis, release and uptake of dopamine in the basal ganglia circuitry of the brain of CKD mice, and the resultant motor abnormalities. Further, the dopamine metabolism may also be affected by anaemia, iron and erythropoietin deficiency, hyperphosphatemia, calcium dysregulation and hyperkalemia-induced alterations in the axonal membrane potential, which have been implicated in human subjects^47,73,74^, and are prevalent in adenine model of CKD as well^50,51,56,59,60^.

The CKD mice had a poor cognitive function, both long-term and short-term, as revealed by the reduced discrimination index in the OLM and ORM tests (Fig. 2C,D). Since cognitive decline is attributed to cholinergic deficiency in brain^26,27,75,76^, we thought it prudent to assess the activity of the enzyme AChE in the brain of CKD mice. AChE plays a crucial role in cholinergic neurotransmitter systems, and is responsible for terminating the nerve impulses at cholinergic synapses by splitting the neurotransmitter acetylcholine into choline and acetate^77^. Histoenzymological study (Fig. 6A–L) as well as estimation from the brain tissue homogenate (Fig. 6M) revealed a global decrease in the activity of AChE in the brain of the CKD mice (Fig. 6). Diminished AChE activity was previously reported in the hypothalamus of rats with CKD^78^. Decrease in the activity of AChE in cortex and hippocampus of brain have been implicated in dementia and cognitive impairment in Alzheimer’s disease (AD) patients^26,27,75,76^, which may thus explain the mechanism underlying the cognitive decline in CKD. Further, Golgi staining revealed decrease in neuronal arborisation in the hippocampus (Fig. 8H), and dendritic spine density in the cortex and hippocampus (Fig. 8G) of the CKD mice. Dendritic spines play important role in neuronal transmission as these receive excitatory inputs^79^, and thus loss of spine density is implicated in neurological complications^79–81^. Since dendritic atrophy and loss of spine density in the cortex and hippocampus are implicated in cognitive deficits^82,83^, we hereby hypothesize the same in the observed cognitive decline in the CKD mice. Further analysis revealed alteration in SOD (Fig. 12A) and Catalase activities (Fig. 12B), astrocytosis (Fig. 13), increase in NOS-active neurons (Fig. 14), and mitochondrial dysfunctions (Figs 9, 10 and 11) in the cortex; and altered SOD (Fig. 12A) and Catalase (Fig. 12B) activities, and astrocytosis (Fig. 13) in the hippocampus of the brain of CKD mice. These factors are hereby speculated to be the underlying pathophysiologies for the observed loss of AChE activity, dendritic atrophy and loss of spines in the cortex and hippocampus of the brain of CKD mice (Figs 6 and 8). Other factors which may cause the dendritic damages include disruption of blood brain barrier, glutamate-mediated excitotoxicity, hypercalcemia, hyperkalemia and uremic neurotoxins^38,84–87^. In addition to the loss of AChE activity and dendritic damages (as revealed in the present study), other factors which may contribute to the cognitive impairment in the CKD include hyperuricemia, hyperkalemia, hypercalcemia, anaemia, hyperparathyroidism, hyperhomocysteinemia, inflammation, and the traditional risk factors like endothelial dysfunction, vascular damages, diabetes, hypertension and silent brain infarct^16–18,47,88–92^, which are prevalent in rodent model of adenine-induced CKD as well^51,56,59,60^. Based on computational modelling, we have recently demonstrated that disturbances in purine nucleotide metabolism, prevalent in CKD patients^93^ as well as adenine model^50,55^, as a risk factor for cognitive decline, mediated by diminished AChE activity^94^.

The CKD mice exhibited depression-like behaviour with higher immobility time in the forced swim test (Fig. 2B), which is consistent with previous reports in animal models^23,52^. Depression and anxiety are among common neurological complications in CKD patients^16,19,95^. Since cholinergic deficiency has been implicated in causing anxiety and depression^96,97^, the depression-like behaviour observed in the CKD mice may also be attributed to the cholinergic deficiency observed in the present study (Fig. 6). Further, loss of dendritic spine density and dendritic atrophy (Fig. 8), astrocytosis (Fig. 13) and calcium dysregulation in the cortex and hippocampus of the CKD mice may contribute to the observed depressive behaviour, since these inflammatory factors along with other associated pathophysiologies, which are prevalent in adenine model of CKD as well^56,60,98^, have been implicated in causing depression^99–109^.

Unlike the renal uroliths, histological studies using Nissl staining (Fig. 4) and Picro-Sirius red staining (Fig. 5) did not reveal any deposition of crystals of adenine and 2,8-dihydroxyadenine in any of the brain regions of the CKD mice, which is consistent with our previous report^23^. This demonstrates that the biochemical and histopathological changes observed in the brain of CKD mice were caused due to CKD, and not as a direct consequence of high adenine diet or hyperuricemia. However, since high adenine diet leads to the development of CKD^23,49–62^ with neurological complications^23,52^ in animal models, including biochemical and histopathological changes (as revealed in the present study), it is highly recommended that the CKD patients, and those who are at higher risk of developing CKD, should lessen dietary intake of adenine, and should manage uric acid level.

To investigate whether the observed changes in the brain of CKD mice were due to high adenine diet and/or the resultant hyperuricemia, or due broadly to CKD, a set of mice were transferred to standard feed post 4 weeks treatment (with high adenine diet) to facilitate wash-out of adenine and uric acid. The mice were then sacrificed on 35^th^ day, and AChE activity assays were performed in different brain regions. The results indicate that the activity of the enzyme did not ameliorate, and the diminished AChE activity persisted significantly in the cortex, striatum, hippocampus, and substantia nigra of CKD mice by 46.74%, 24.43%, 43.87% and 25.58% respectively, compared to the control (Supplementary Figs S1 & S2). Thus, it is argued that the decrease in the activity of the enzyme was not a direct consequence of adenine-rich diet or hyperuricemia, and rather due to CKD as a whole. However, the involvement of the uremic toxins on the observed neurological changes in the CKD mice should not be ignored. Guanidinosuccinic acid, a uremic toxin known to be elevated in CKD patients^110^ as well as adenine model of CKD^50^, has been reported to cause clonic seizures, status epilepticus and damages to CA1 hippocampal neurons^84^. However, the effects of other uremic toxins on brain functions with respect to possible histopathological and neurochemical changes have not been elucidated yet. Although middle molecules (mol. wt. 300–12,000 Da) have been postulated to be uremic neurotoxins^86^, limited evidence exists to ascertain the same^87^. Further, disturbances in sodium, potassium and calcium levels in the brain, as well as peripheral nervous system, have been suggested to impair neurotransmission^85,90,91^, and thereby contribute to the neurological complications in CKD. These disturbances are prevalent in the adenine model of CKD also^51,56,59,60^. Thus, further studies are warranted for investigating the role of individual uremic toxins in causing the neurological complications.

Taken together, the present study puts forward a comprehensive insight into the histopathological and neurochemical alterations occurring in the brain of CKD mice, and thus the pathophysiological basis underlying the development of neurological complications in CKD. Since CKD is a growing global health burden, and leads to neurological complications thereby affecting quality of life, adherence to treatment regimens, sleep and mood disturbances, in addition to psycho-motor and cognitive functional decline, the present findings are vital in understanding the mechanisms underlying the complications. Thus, the present study has therapeutic implications in the management of the neurological complications in CKD.

## Supplementary information

Supplementary Information
