# Supplementary material for: Neurological sequel of chronic kidney disease: From diminished Acetylcholinesterase activity to mitochondrial dysfunctions, oxidative stress and inflammation in mice brain
Source: Sci Rep. 2019 Feb 28;9:3097. doi: 10.1038/s41598-018-37935-3 (PMC6395638; doi:10.1038/s41598-018-37935-3)
Supplement: Supplementary file 1 — Supplementary Information [file 41598_2018_37935_MOESM1_ESM.doc]

**Neurological sequel of chronic kidney disease: From diminished Acetylcholinesterase activity, to mitochondrial dysfunctions, oxidative stress and inflammation in mice brain**

Muhammed Khairujjaman Mazumder^1^, Rajib Paul^1,2^, Pallab Bhattacharya^3^ & Anupom Borah^1#^

*^1^Cellular and Molecular Neurobiology Laboratory, Department of Life Science and Bioinformatics, Assam University, Silchar-788011, Assam, India*

*^2^Department of Zoology, Pandit Deendayal Upadhyaya Adarsha Mahavidyalaya (PDUAM), Eraligool-788723, Karimganj, Assam, India*

*^3^Department of Pharmacology and Toxicology, National Institute of Pharmaceutical Education and Research (NIPER)-Ahmedabad, Gandhinagar-382355, Gujarat, India*

#Correspondence and requests for materials should be addressed to A. B. (anupomborahh@gmail.com)

**Rationale and Methodology.** To investigate whether the observed neurological changes in the CKD mice were due to the effect of high adenine diet and/or the resultant hyperuricemia, or due broadly to CKD, a set of mice were shifted to standard feed post 4 weeks treatment with high adenine diet. The mice were maintained with standard feed for 7 days to facilitate wash-out of adenine and uric acid, and sacrificed on 35^th^ day. The assay for Acetylcholinesterase (AChE) activity was performed in brain slices as well as from tissue homogenates.

**AChE histology.** AChE activity was estimated from brain tissue sections, following Paul and Borah^34^. Briefly, 20 µm thick sections passing through cortex, striatum, hippocampus and substantia nigra were taken on poly-L-Lysine coated slides using cryostat. The sections were processed for AChE activity as described earlier.

**AChE by Ellman’s method.** The AChE activity was determined from tissue homogenates of the intended brain regions (cortex, striatum, hippocampus and substantia nigra), following Ellman’s method^35^ as described earlier.

**Results.** The histoenzymological study of the AChE activity in the different brain regions of the CKD mice revealed a marked visible decrease in the colour intensity, compared to the control mice (Supplementary Fig. 1). Moreover, estimation of the activity of the enzyme from homogenates from these brain regions revealed that the activity of the enzyme (in mu/mg protein, represented as Mean ± S.D.) in the cortex, striatum, hippocampus, and substantia nigra of the control mice was 13.05±2.05, 38.05±3.88, 24.46±2.34, and 32.54±3.76 respectively, while in the CKD mice the same was 6.95±0.82, 28.75±2.97, 13.73±2.1, and 24.22±2.6 respectively. Thus, there was a significant decrease (at p ≤ 0.05, n=6) in the AChE activity in cortex, striatum, hippocampus, and substantia nigra of CKD mice by 46.74%, 24.43%, 43.87% and 25.58% respectively, compared to the control mice (Supplementary Fig. 2). When estimated on 28 days of high adenine diet, the AChE activity was found to be decreased significantly by 43.97%, 27.07%, 41.09%, and 28.56% in cortex, striatum, hippocampus and substantia nigra regions respectively, in CKD mice compared to control (Fig. 4). This clearly indicated that despite withdrawal of the adenine rich diet, the activity of AChE did not ameliorate, and thus it is argued that the decrease in the activity of the enzyme was not a direct consequence of adenine-rich diet or hyperuricemia, rather broadly due to CKD.


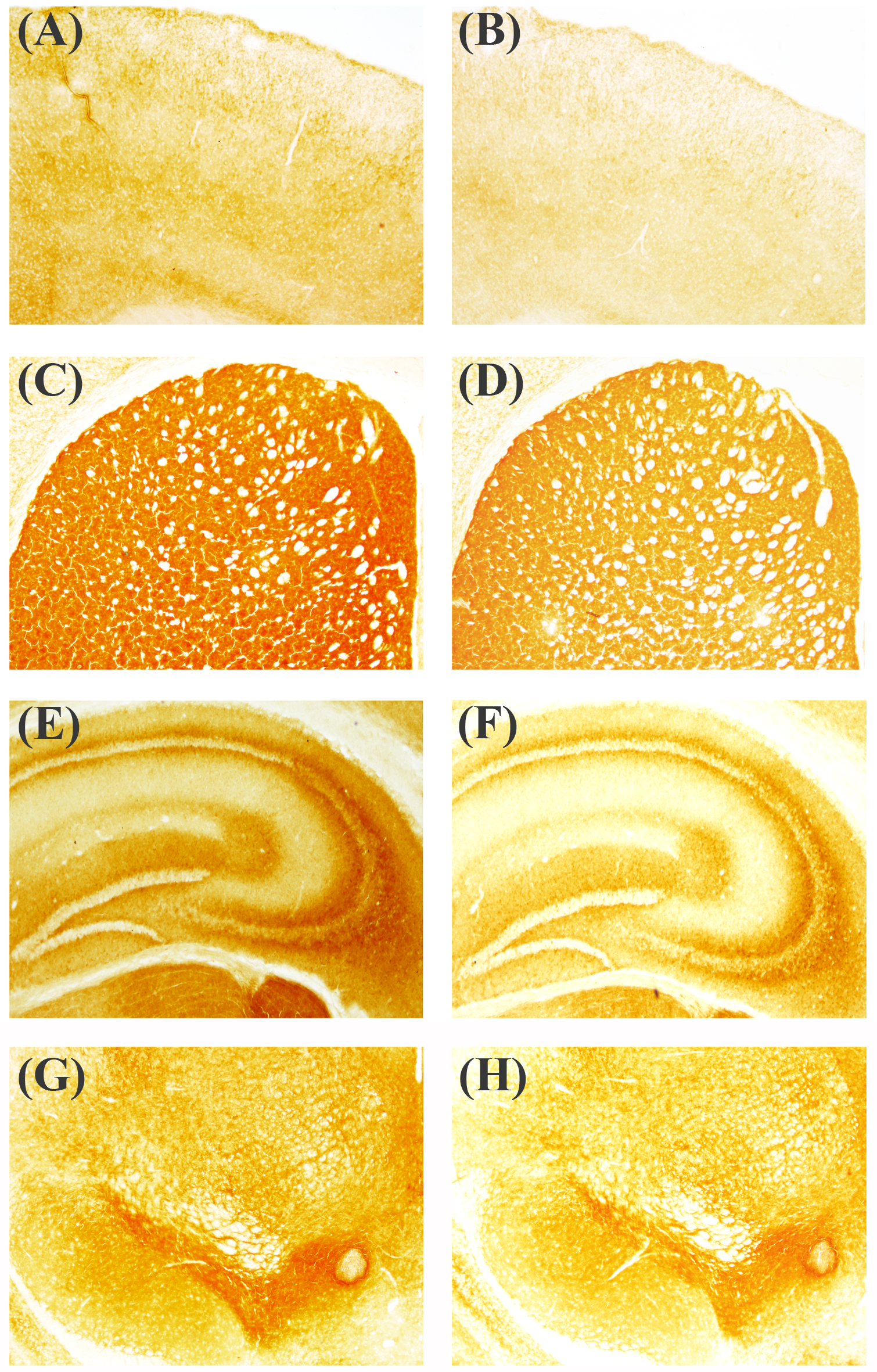


**Figure S1:** Acetylcholinesterase (AChE) activity in different brain regions of control (CS) and chronic kidney disease (CKD) mice. Representative sections of AChE histoenzymology in different brain regions: (**A,B**) cortex, (**C,D**) striatum (NCP), (**E,F**) hippocampus and (**G,H**) substantia nigra. The CS mice received standard diet while CKD mice were given adenine at the dose of 0.3% w/w mixed with standard feed for 28 days. AChE activity was tested 7 days after the last dose of high adenine diet. For these 7 days post treatment, mice were maintained with standard feed, without supplementation of adenine. 20 µm thick sections from glycerol perfused brain were stained for AChE histoenzymology. The activity of the enzyme AChE, indicated by colour intensity, was found to be decreased in all the brain regions of CKD mice (**B,D,F,H**), compared to CS (**A,C,E,G**). Photographs were taken under bright field illumination at 4 × magnification.


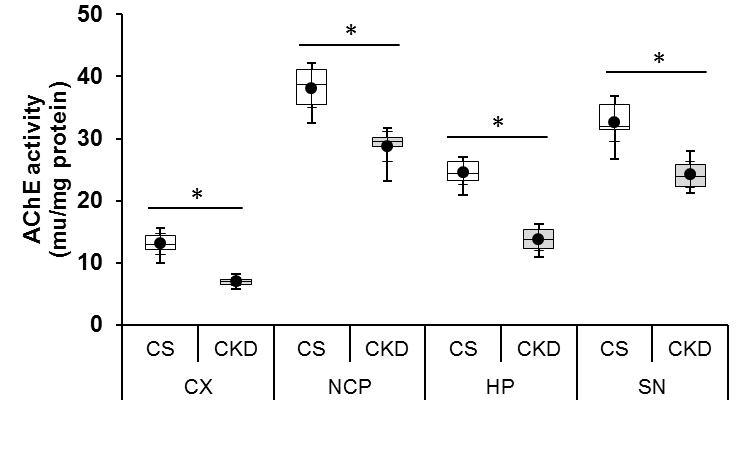


**Figure S2:** Distribution of AChE activity values in control (CS) and chronic kidney disease (CKD) mice shown as box plot. The activity of AChE (in mu/mg protein) in the brain tissue homogenates from the intended brain regions of CS and CKD mice was estimated following Ellman method. Animals were sacrificed 7 days after the last dose of high adenine diet. The box extends from 25^th^ to 75^th^ percentile, the line at the middle of the box represent median, and the whiskers delimit minimum to maximum values. The dot within the box represents the mean of the group with 95% confidence interval denoted by error bars. *p ≤ 0.05 as compared to control was considered statistically significant (n = 6). Data represents Mean±S.D.

CX = cortex; NCP = striatum; HP = hippocampus; SN = substantia nigra.

**Supplementary Table 1:** Table showing body weight, scores of behavioural tests and hematological parameters in control (CS) and chronic kidney disease (CKD) mice, showing p-values. The body weights were taken on every alternate day. *P* - values ≤ 0.05 was considered significant (*), while others were considered not significant (NS), compared to CS.

| **Experiment** | | **Tested values / scores**  **(Mean ± S.D.)** | | **p-value** | **Significance** |
| --- | --- | --- | --- | --- | --- |
|  |  | **CS** | **CKD** |  |  |
| Body Weight (in gram)  (Taken on alternate days) | 0 | 25.96±0.62 | 26.18±0.67 | 0.5598 | NS |
|  | 2 | 26.14±0.559 | 26.01±1.55 | 0.8509 | NS |
|  | 4 | 26.8±0.596 | 26.17±1.54 | 0.3762 | NS |
|  | 6 | 27.21±1.263 | 26.18±1.12 | 0.1683 | NS |
|  | 8 | 27.39±1.592 | 25.40±2.05 | 0.0895 | NS |
|  | 10 | 27.28±1.68 | 25.01±2.81 | 0.1210 | NS |
|  | 12 | 28.33±1.776 | 25.13±2.67 | 0.0341 | * |
|  | 14 | 28.98±1.06 | 22.08±2.31 | <0.0001 | * |
|  | 16 | 28.78±1.124 | 22.25±2.18 | <0.0001 | * |
|  | 18 | 29.72±1.075 | 20.60±1.88 | <0.0001 | * |
|  | 20 | 30.21±1.544 | 20.03±2.07 | <0.0001 | * |
|  | 22 | 30.96±0.968 | 21.09±1.22 | <0.0001 | * |
|  | 24 | 31.08±1.295 | 20.64±1.68 | <0.0001 | * |
|  | 26 | 31.06±1.389 | 19.15±1.80 | <0.0001 | * |
|  | 28 | 31.92±1.5 | 18.63±1.56 | <0.0001 | * |
| % Change in body weight  (compared to initial body weight) | | 22.92±3.65 | -28.90±4.69 | <0.0001 | * |
|  | | | | | |
| Swim test  (Total Swim score; in sec) | | 26.33±0.82 | 21.17±1.60 | <0.0001 | * |
| Forced Swim test  (Total Immobility time; in sec) | | 43.67± 7.12 | 83.17±7.25 | <0.0001 | * |
| Object Location test  (% Discrimination Index) | | 46.00±7.13 | 30.17±4.07 | 0.0008 | * |
| Object Recognition Test  (% Discrimination Index) | | 41.50±5.65 | 19.75±8.07 | 0.0003 | * |
|  | | | | | |
| Urea (in mg/dL) | | 44.5±8.50 | 163.2±9.64 | <0.0001 | * |
| Creatinine (in mg/dL) | | 0.50±0.14 | 2.42±0.42 | <0.0001 | * |
| Uric acid (in mg/dL) | | 2.92±0.86 | 6.62±1.06 | <0.0001 | * |

**Supplementary Table 2:** Table showing data of histological and biochemical parameters in control (CS) and chronic kidney disease (CKD) mice. The activities of AChE, mitochondrial complex-I, SOD and Catalase, and extent of lipid peroxidation were tested from brain tissue homogenates using spectrophotometric methods. The activities of mitochondrial complex-II and complex-III, and TH-immunoreactivity in NCP were estimated following densitometric analysis of the images of the histoenzymological studies to determine optical density. The dopaminergic neuronal count in the SN, and neuronal arborisation and dendritic spine density in the CX and HP were performed directly from the images of the respective brain regions, obtained following TH-immunoreactivity and Rapid Golgi staining respectively. *P* - values ≤ 0.05 was considered significant (*), while others were considered not significant (NS), compared to CS.

| **Experiment** | **Brain regions** | **Test values (Mean ± SD)** | | **p-Value** | **Significance** |
| --- | --- | --- | --- | --- | --- |
|  |  | **CS** | **CKD** |  |  |
| AChE activity  (in mu/mg protein) | PFC | 28.23±2.41 | 17.57±3.76 | 0.0002 | * |
|  | CC | 13.01±1.99 | 7.29±1.27 | 0.0001 | * |
|  | NCP | 39.34±4.42 | 28.69±3.10 | 0.0007 | * |
|  | AMG | 35.67±5.55 | 21.01±3.28 | 0.0002 | * |
|  | HP | 25.17±2.42 | 14.37±2.35 | <0.0001 | * |
|  | SN | 33.61±3.69 | 24.01±2.45 | 0.0003 | * |
|  | | | | | |
| TH-Immunoreactivity  (Optical Density) | NCP | 0.349±0.027 | 0.321±0.024 | 0.0841 | NS |
| TH-positive neuronal count | SN | 246.33±12.71 | 231.33±13.43 | 0.4198 | NS |
|  | | | | | |
| Rapid Golgi staining  (Neuronal arborisation) | HP | 4.4±1.07 | 3.4±0.57 | 0.04803 | * |
| Rapid Golgi staining  (No. of spine/10µM) | CX | 6.2±1.03 | 4.9±0.99 | 0.0102 | * |
|  | HP | 7.1±1.19 | 5.8±0.79 | 0.0102 | * |
|  | | | | | |
| Mitochondrial complex-I activity  (NADH oxidized/min/mg protein) | CX | 123.12±16.81 | 99.78±17.08 | 0.0382 | * |
|  | NCP | 101.34±11.95 | 83.78±9.49 | 0.0412 | * |
|  | HP | 78.67±9.17 | 71.34±8.20 | 0.1749 | NS |
|  | SN | 119.31±13.39 | 105.78±12.99 | 0.1059 | NS |
|  | | | | | |
| Mitochondrial complex-II activity  (Optical density) | CX | 0.096±0.010 | 0.066±0.009 | 0.0002 | * |
|  | NCP | 0.090±0.006 | 0.071±0.006 | 0.0002 | * |
|  | HP | 0.102±0.009 | 0.093±0.009 | 0.0954 | NS |
|  | SN | 0.069±0.004 | 0.063±0.010 | 0.1941 | NS |
|  | | | | | |
| Mitochondrial complex-III activity  (Optical density) | CX | 0.226±0.036 | 0.183±0.024 | 0.0328 | * |
|  | NCP | 0.228±0.024 | 0.188±0.028 | 0.0219 | * |
|  | HP | 0.246±0.026 | 0.229±0.026 | 0.2902 | NS |
|  | SN | 0.301±0.024 | 0.290±0.023 | 0.5078 | NS |
|  | | | | | |
| Superoxide dismutase activity  (in Units/mg protein) | CX | 2.11±0.24 | 2.97±0.50 | 0.0033 | * |
|  | NCP | 1.45±0.19 | 1.89±0.41 | 0.0359 | * |
|  | HP | 2.43±0.54 | 3.21±0.63 | 0.0469 | * |
|  | SN | 0.83±0.23 | 1.22±0.23 | 0.0172 | * |
|  | | | | | |
| Catalase activity  (in Abs/min/mg protein) | CX | 3.11±0.24 | 2.57±0.41 | 0.0464 | * |
|  | NCP | 2.45±0.15 | 1.89±0.38 | 0.0471 | * |
|  | HP | 3.11±0.53 | 2.35±0.25 | 0.0453 | * |
|  | SN | 2.33±0.20 | 1.78±0.34 | 0.0493 | * |
|  | | | | | |
| Lipid peroxidation  (in nmol/mg protein) | CX | 4.35±0.62 | 5.56±0.81 | 0.0421 | * |
|  | NCP | 6.57±1.09 | 8.32±0.58 | 0.0472 | * |
|  | HP | 7.01±1.75 | 7.78±1.80 | 0.5601 | NS |
|  | SN | 6.53±1.23 | 6.89±1.25 | 0.6957 | NS |

*Abbreviations: CS, Control mice; CKD, Chronic Kidney Disease mice; PFC, Pre-Frontal Cortex; CC, Cerebral Cortex; NCP, Striatum; AMG, Amygdala; HP, Hippocampus; SN, Substantia nigra; AChE, Acetylcholinesterase; TH, Tyrosine hydroxylase.*
